# Stoichiometric multitrophic networks reveal significance of land-sea interaction to ecosystem function in a subtropical nutrient-poor bight, South Africa

**DOI:** 10.1371/journal.pone.0210295

**Published:** 2019-01-07

**Authors:** Ursula M. Scharler, Morag J. Ayers

**Affiliations:** School of Life Sciences, University of KwaZulu-Natal, Durban, South Africa; Swedish University of Agricultural Sciences and Swedish Institute for the Marine Environment, University of Gothenburg, SWEDEN

## Abstract

Nearshore marine ecosystems can benefit from their interaction with adjacent ecosystems, especially if they alleviate nutrient limitations in nutrient poor areas. This was the case in our oligo- to mesotrophic study area, the KwaZulu-Natal Bight on the South African subtropical east coast, which is bordered by the Agulhas current. We built stoichiometric, multitrophic ecosystem networks depicting biomass and material flows of carbon, nitrogen and phosphorus in three subsystems of the bight. The networks were analysed to investigate whether the southern, middle and northern bight function similarly in terms of their productivity, transfer efficiency between trophic levels, material cycling, and nutrient limitations. The middle region of the bight was clearly influenced by nutrient additions from the Thukela River, as it had the highest ecosystem productivity, lower transfer efficiencies and degree of cycling. Most nodes in the networks were limited by phosphorus, followed by nitrogen. The middle region adjacent to the Thukela River showed a lower proportion of P limitation especially in summer. Interestingly, there was a clear distinction in sensitivities to nutrient limitations between lower and higher trophic level organisms. This is a reflection of their discrepant nutrient turnover times that are either higher, or lower, than that of the systems, and which might provide a balance to the system through this antagonistic influence. Furthermore, by tracking the stoichiometry through entire food webs it appeared how important the role of lower trophic level organisms was to regulate stoichiometry to more suitable ratios for higher trophic level requirements. Although we gained good insight into the behaviour of the three subsystems in the KZN Bight and the role of terrestrial influence on their functioning, a merged approach of incorporating data on metabolic constraints derived from experiments could further improve the representativeness of multitrophic stoichiometric ecosystem networks.

## Introduction

Coastal areas, including estuaries, wetlands and the nearshore are among the most productive habitats on Earth. These habitats do not function in isolation, but are interconnected through chemical and biological interchange between them [1,2]. This means that realms may depend on each other for their sustained productivity [3], and an understanding of the interconnectivity's influence on ecosystem functioning is therefore important to their management and conservation [4,5]. How this interconnectivity of habitats is reflected in energy and nutrient pathways and affects multitrophic ecosystem function has hardly been explored [4,6].

Riverine input to the nearshore is one form of habitat connectivity that has impacts in coastal oceans. River plumes, for instance, are a distinct feature of many coastlines. Kang et al. [7] estimated that the world's largest 19 rivers have a mean annual plume area that is > 14% of the world's continental shelf area. Although not all coasts feature large rivers, many coastal areas have experienced negative impacts from riverine runoff due to nutrient outflow, consequent blooms followed by low oxygen conditions that may lead to the formation of dead zones with wider reaching influences on food webs [8,9]. Concentrations of nutrients, chlorophyll-a and anthropogenically derived chemicals (e.g. pesticides) are often found in higher concentrations within plumes when present [10]. However, on oligo- to mesotrophic coasts, the terrigenous runoff may actually play a highly significant role in stimulating nearshore production [11], noticeable especially in areas featuring strong seasonal signals for river flow.

The functioning of an ecosystem, from a nutrient point of view, depends not only on its sources, but how the system's organisms consume, transform, recycle and excrete nutrients, and use them for growth and reproduction [12,13]. Within an ecosystem, carbon [C], nitrogen [N] and phosphorus [P] are important macronutrients in processes across various levels of organisation from the cellular to the ecosystem level. For instance, the growth of individual organisms can be limited by low nutrient concentrations which can affect population dynamics [14] and interspecific interactions [15,16] within communities. Nutrient limitations can also affect key ecosystem processes such as nutrient cycling [17–20] and ecosystem structure [21]. Nutrient limitation at the levels of organisms, populations and ecosystems could thus extend to fisheries yields, especially that of top predators affecting fisheries and food security in nutrient-poor coastal regions in the tropics and subtropics. In areas where riverine outflow is a major nutrient source, it has been shown to increase production of lower trophic levels and that of target and forage fish species [22–24]. Productivity in the Gulf of Aden decreased after reduced river flow following the construction of the Aswan Dam on the Nile River [25], and increased in the region following higher nutrient delivery [26]. Such autochthonous sources to nutrient-poor systems can be important, but may also be responsible for altering elemental composition and stoichiometry, of autotrophs and heterotrophs [27].

Freshwater systems [28,29] have received more attention in comparison to marine ecosystems in terms of the stoichiometry of populations and communities. In the latter, most studies are restricted to phytoplankton or organic matter stoichiometry [30–32], and other investigations are often confined to single groupings [e.g. benthos [33], Crustacea [34] or Teleostei [35]. Few ecosystem-level studies have incorporated stoichiometric data [20,36–39], and to our knowledge only one of these ensured adherence to node and flow stoichiometry during multitrophic network construction and mass balancing [20]. A more comprehensive understanding of nutrient content and multitrophic stoichiometry in an ecosystem can be particularly useful for interpreting the structure and functioning of food webs. Our study area, the KwaZulu-Natal Bight (KZN Bight) on the subtropical east coast of South Africa, relies on seasonal or intermittent oceanic and riverine nutrient sources. Previous studies have investigated various aspects of the physical and biological oceanography of the region, as well as effects of riverine input from the Thukela river on selected biota in the KZN Bight [40,41].

Considering issues such as increased river water abstraction for agriculture and industry, and climate change affecting rainfall and food security, it is important to understand the role of riverine nutrient input to the productivity and function of coastal marine ecosystems. In this study, a particular emphasis was placed on the spatial variability of various abiotic components and biotic communities in three areas of the KZN Bight, which represent its northern, middle and southern regions. We aimed to investigate nearshore multi-nutrient food webs with particular reference to riverine influence on the heterogeneity of the bight ecosystem in terms of its productivity, nutrient transfer efficiency, stoichiometry and nutrient limitations.

## Methods

### Study areas

**Fig 1:**
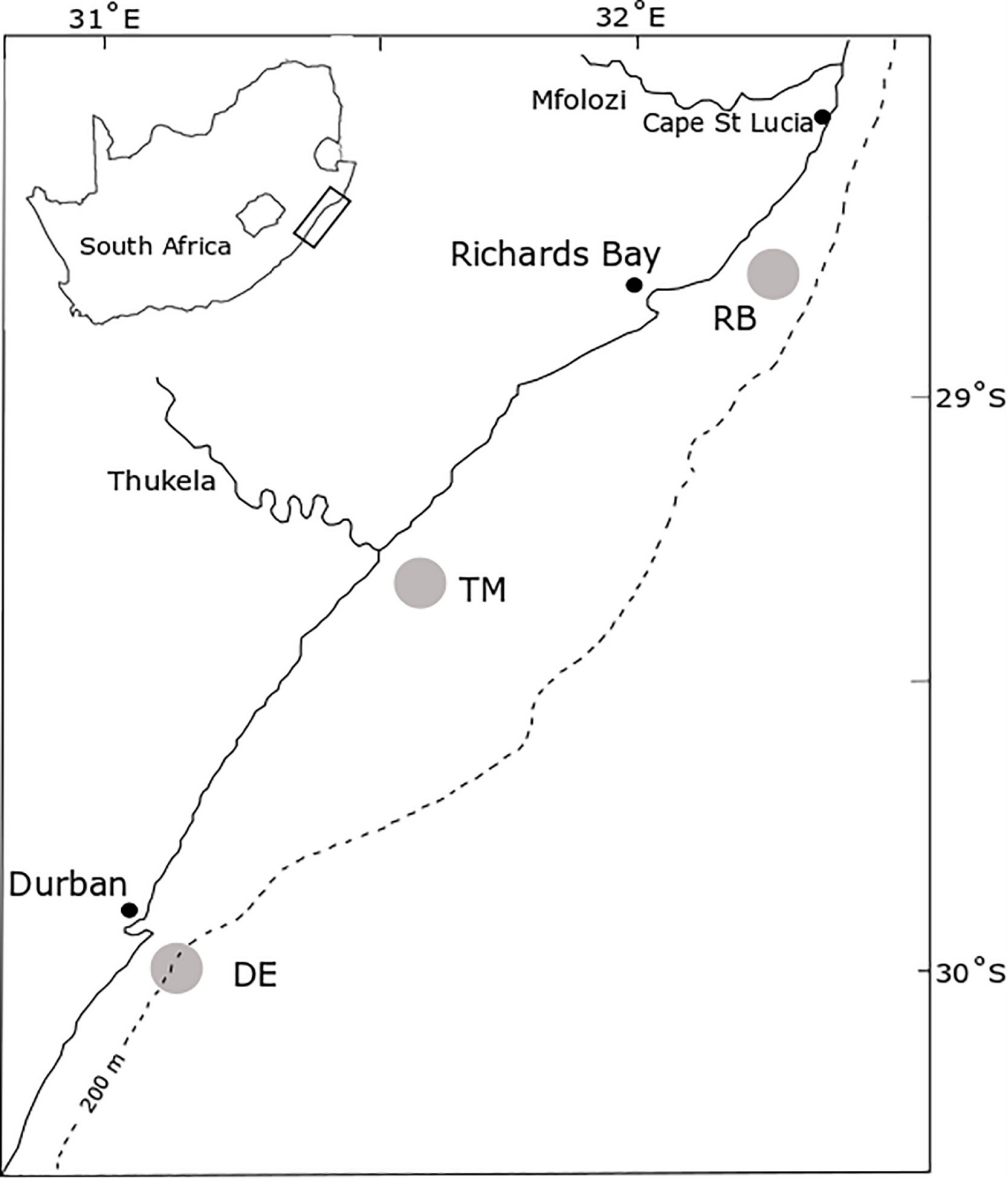
The KwaZulu-Natal Bight and location of subsystems. DE: Durban Eddy, TM: Thukela Mouth, RB: Richards Bay.

The KZN Bight extends from Durban in the south (29.8587° S, 31.0218° E) to Cape St. Lucia in the north (28.5151° S, 32.3995° E) and to the edge of the Agulhas current at the 200m isobath (Fig 1) [42]. Three subsystems were chosen for analyses based on sampling sites during the African Coelacanth Ecosystem Programme (ACEP II) research cruises in summer (February) and winter (August) 2010 [43]. These were based on hypothesised locations of the three major nutrient sources to the bight [southern eddy, central river outflow, northern upwelling]. The “Durban Eddy” subsystem (DE) is located at the 200m isobath in the southern area of the bight (Fig 1). The “Thukela River Mouth” subsystem (TM) is located between the 30m and 40m isobaths in the central area of the bight (Fig 1). Lastly, the “Richards Bay” subsystem [RB] is located between the 30m and 40m isobaths in the northern area of the bight (Fig 1).

### Modelling approach

The spatial heterogeneity of ecosystem function within the KZN bight was investigated through multitrophic stoichiometric networks depicting biomass and carbon (C), nitrogen (N) and phosphorus (P) exchanges within the ecosystem and across the system boundary. Networks were constructed for each subsystem representing the southern (DE), middle (TM) and northern (RB) bight. For each of the three areas, a network representing the summer season and winter season were constructed, resulting in a total of 18 networks (three areas, two seasons, three nutrients).

The carbon networks were partially based on previously established networks representing the KZN Bight [44] and Thukela area [45]. For this study, networks were constructed for all three areas of the bight, and species representing the three areas were aggregated into the same functional groups for all networks (S1 Table) [40,46] to account for the influence of node aggregation on ENA metrics [37,47,48]. Detritus was partitioned into suspended particulate organic matter (POM), sediment POM, dissolved organic matter (DOM) and dissolved inorganic matter (DIM). All data sources for all nodes are outlined in S2, S3 and S4 Tables. Cetaceans were not included in the DE network because resident dolphin species, which dominate the biomass of this group, do not occur beyond the 50m isobath [49,50]. Other data used for network construction were calculated from measurements taken during the two ACEP II cruises and related demersal trawls in 2010, or from literature sources (see Data sources section).

Carbon networks were constructed and parameterised first using Ecopath with Ecosim software, version 6 [51]. Input parameters used to construct Ecopath networks include diet composition, biomass (gC m^-2^), production/biomass ratio (P/B, year^-1^), consumption/biomass ratio (Q/B, year^-1^) and ecotrophic efficiency (EE, proportion) which represents the proportion of production utilised in the system. If one of either B, P/B, Q/B or EE is unknown, it is estimated using two mass-balance equations which ensure the inflows into each group in the system are equal to its outflows [52]. Nitrogen and phosphorus networks were constructed using data from carbon networks and C:N, C:P and N:P ratios from [40] or literature (see 2.3 Data Sources). From these stoichiometric, mass-balanced networks, nutrient transfer efficiencies and recycling, nutrient limitations for nodes and limiting flows were calculated (see 2.4 Network Analysis Methodology), to illustrate differences and similarities in the three subsections of the KZN Bight, and link them to influences of adjacent ecosystems.

### Construction of multitrophic stoichiometric networks

#### Data sources

Carbon biomasses of most model groups were measured from samples collected during the 2010 ACEP II research cruises [40]. Other basic input data, needed to construct Ecopath networks (P/B, Q/B, EE) were collected from published and grey literature (S2 Table). Exceptions to the latter were the P/B ratios of diatoms, flagellates and of prawn and shrimp. Ratios for diatoms and flagellates were calculated from biomass and production measurements in each subsystem [53]. Due to a lack of production measurements during the August [winter] cruise, P/B ratios were based on measurements from the February [summer] cruise. Diatom P/B was 199 y^-1^, 242 y^-1^ and 490 y^-1^ in the DE, TM and RB subsystems respectively. Flagellate P/B was 208 y^-1^ in the DE subsystem, 406 y^-1^ in the TM subsystem and 410 y^-1^ in the RB subsystem. In the DE networks, at the 200m isobath, a P/B of 2.5 y^-1^ was used for prawn and shrimp based on deep-water prawns [54], while in the TM and RB networks, which represent sites between 30m and 40m isobaths, a P/B of 2.73 y^-1^ was used based on shallow-water prawns [54].

Diet compositions in the networks were derived from an isotope study of the region [55,56], and from literature (S3 Table). The fate of biomass not consumed in the systems was assigned to the various [non-living] abiotic groups (S2 Table).

Imports in the form of suspended POC, DOC and DIC were included in the TM and RB networks due to significant river outflow to the study areas. In the TM networks, suspended POC import was calculated using a sediment concentration for the Thukela River of 4.28g L^-1^, the area of the Thukela mudbanks (561 km^2^) [45] and flow rates based on a January flow rate of 429 m^3^ s^-1^ and a June flow rate of 25 m^3^ s^-1^ [57]. Values of DOC import were calculated based on an average global estimate for rivers of 6 mg L^-1^ [58], and the flow rates and area mentioned above. DIC imports for the TM networks were calculated using DIN values from the Thukela River estuary (see below) and a DIC:DIN ratio of 8.2 [59]. In the RB networks, a sediment concentration of 0.425g L^-1^ was calculated based on a sediment yield for the Mfolozi River of 6.8x10^5^ t y^-1^ and a mean annual flow of 1.6x10^12^ L y^-1^ [60]. A POC concentration of 2.57x10^10^ g y^-1^ was calculated using the sediment concentration and the assumption that POC was 8.4% of TSS [59]. DOC imports were calculated based on an average global estimate of 6 mg L^-1^ [59], a January flow rate of 6x10^10^ L and a June flow rate of 6x10^9^ L for the Mfolozi River [61]. A summary of imports can be found in Table 1.

**Table 1:** Detrital imports [g m^-2^ y^-1^] into the Thukela Mouth and Richards Bay networks.

		Thukela Mouth		Richards Bay	
Detritus group		Summer	Winter	Summer	Winter
Suspended POM	C	8 671 358	501 964	45 900	4 582
	N	1 057 483	61 215	5 598	559
	P	433 135	25 073	2 293	229
DOM	C	144 789	8 381	7 701	770
	N	7 239	419	385	39
	P	144	8	8	1
DIM	C	62 332	1 306	3 315	120
	N	7 601	159	404	15
	P	531	17	28	2

Biomasses, imports and exports in the nitrogen and phosphorus networks were calculated based on the corresponding carbon data for each network and C:N and C:P molar ratios measured from samples taken during the two cruises and demersal trawls [40] or from literature (S4 Table). Seasonal DIN and DIP imports were calculated for the TM and RB networks using the flow rates and area mentioned in the carbon data sources above and nitrate and phosphate concentrations from the Thukela River [57] (Table 1). The C, N and P biomass for all 18 networks is shown in S5 Table.

#### Parameterisation of carbon networks

Six carbon networks representing each subsystem in summer 2010 and winter 2010 were constructed. Initially, none of the six carbon networks were mass-balanced. The proportion of the production utilised in the system (EE) was higher than one for many groups because outflows, in the form of predation, were larger than inflows. To balance the networks, biomass data of groups which were undersampled by the ACEP II trawling gear (other cephalopods, cuttlefish, molluscs, echinoderms, large suspension feeders) [46] were removed from one or all of the carbon networks and instead estimated using an EE of 0.95 [62] in Ecopath. The proportion of cannibalism was decreased (as suggested by [62]) in large sharks and replaced with diet imported from outside the subsystem. Diet compositions were adjusted until all groups were balanced and at EE<1.

Thereafter, C networks were extracted from Ecopath to include DIC, and build the corresponding N and P networks. Ecopath does not include the uptake of DIM by primary producers. After the networks were extracted from Ecopath, DIC was added to the carbon networks, calculated using DIN measured in each subsystem during ACEP II cruises [53] and a C:N ratio of 8.2 [59]. Flows from DIC to primary producer groups were calculated based on demand, so that inflow into each primary producer group balanced outflow. The DIC node was balanced based on demand by adding imports or exports across the system boundary.

#### Nitrogen and phosphorus networks

N and P biomasses were calculated using the stoichiometric ratios for each group (S3 Table). The N and P flows between nodes were calculated by multiplying the carbon flow value by the C:N or C:P ratio of the donor biomass. Thereafter, a layered balancing procedure was used to mass balance the network while simultaneously accounting for the large difference in magnitudes of flows and preserving stoichiometry. One layer comprises all flows with a magnitude of the same power of 10. Layers are balanced one by one while keeping other layers constant, in an iterative fashion until convergence is achieved [20].

### Network analysis methodology

The eighteen networks were analysed using ecological network analysis (ENA) to determine differences in size and functioning between subsystems in terms of carbon, nitrogen and phosphorus and if these differences were associated with riverine nutrient sources. Nutrient limitations, cycling and flow transfer efficiencies across trophic levels were calculated to compare differences in functioning and determine the possible role of riverine nutrient sources.

#### System metrics

Firstly, the size of each system was determined by its activity as the Total System Throughflow (TSTf), which denotes the sum of all nodal inflows, or nodal outflows, including flows across the system boundary:

$$TSTf=\sum_{i,j}T_{ij}+z,\;\mathrm{or}$$ (Eq 1)

$$TSTf=\sum_{i,j}T_{ij}+e+r,$$ (Eq 2)

where *T*_*ij*_ is the flow from node *i* to node *j*, *z* the boundary inflow, and *e* and *r* the export, and respiration flow respectively across the boundary.

Nutrient transport between trophic levels in each subsystem was determined by assigning each node to a trophic level according to its feeding activity [63]. The trophic level of each species was calculated as column sum of the input structure matrix [64]. The individual values of the column represent the proportional feeding activity of a node on various trophic levels. Primary producers and detritus nodes were assumed to have a TL of one. Trophic transfer efficiencies between discrete trophic levels were calculated as the fraction of input passed on to the next level via predation [65].

Nutrient cycling in proportion to the systems size was determined using the Finn Cycling Index (FCI), which measures the fraction of throughput recycled [66,67]. This was calculated as follows:

$$FCI=\sum_{i}\frac{N_{ii}-1}{N_{ii}}T_{i}$$ (Eq 3)

where *T*_*i*_ is the total throughput through group *i* and (*N*_*ii*_ -1) is the throughput through group *i* resulting from cycling.

The R package enaR [68] was used to calculate the above indices.

#### Nutrient limitations of nodes and flows

Firstly, nodal nutrient limitations are calculated *sensu* Liebig's Law of the Minimum [69] from the turnover times of the three elements (C, N, P) in each compartment. Secondly, the identity (C, N or P) of the limiting flow between network components is found. Both are calculated through a biomass inclusive ascendency that is most sensitive to the limiting element [70].

For this study, the biomass inclusive ascendency was calculated in order to take into account the storage of elements [C, N, P] in each compartment of the food web, and the distribution of biomass and flows between pairs of nodes [source, receiver]. Firstly, the law of mass action was applied to estimate the exchanges between nodes, where the sum of biomass of source nodes *i* (*ΣB*_*i*_), and the biomass of sink nodes *j* (*ΣB*_*j*_) reflect the total biomass in the system (*B*_._ = *Σ*_*i*_ *B*_*i*_ = *Σ*_*j*_ *B*_*j*_). *A priori*, mass action estimates the flow leaving node *i* as [B_i_/B.] and the amount entering node *j* as (*B*_*j*_*/B*_._), so that the joint probability of a flow both leaving *i* and entering *j* is (*B*_*i*_*B*_*j*_*/B*.^*2*^). The *a posteriori* conditional probability estimation of material actually leaving node *i* and entering node *j*, i.e. the flow from *i* to *j* (*T*_*ij*_) is defined from observations as *T*_*ij*_*/T*_…_ The subscript '..' describes the summation over *i* and *j*. The information gained by knowing the flows *a posteriori* compared to estimating the exchange by the a priori probability (*B*_*i*_*B*_*j*_*/B*.^*2*^) can be estimated, according to [71], as:

$$I_{B}=\sum_{i,j}\left(\frac{T_{ij}}{T_{..}}\right)\left\{-K\log\left(\frac{B_{i}B_{j}}{B.^{2}}\right)-\left[-K\log\left(\frac{T_{ij}}{T_{..}}\right)\right]\right\}$$ (Eq 4)

or

$$I_{B}=\sum_{i,j}\left(\frac{T_{ij}}{T_{..}}\right)K\log\left(\frac{T_{ij}B.^{2}}{T_{..}B_{i}B_{j}}\right).$$ (Eq 5)

The Kullback divergence, or information (*I*_*B*_) gained by knowing the flows is then scaled by the total system throughput (i.e., K is set equal to *T*_.._) to arrive at the biomass inclusive ascendency *A*_*B*_:

$$A_{B}=T_{..}\sum_{i,j}\frac{T_{ij}}{T_{..}}\log\left(\frac{T_{ij}B.^{2}}{T_{..}B_{i}B_{j}}\right)$$ (Eq 6)

or

$$A_{B}=\sum_{i,j}T_{ij}\log\left(\frac{T_{ij}B.^{2}}{T_{..}B_{i}B_{j}}\right).$$ (Eq 7)

The biomass inclusive ascendency (*A*_*B*_) can be extended to apply to each particular nutrient [C, N or P] of interest, using the formula:

$$A_{B}=\sum_{i,j,k}T_{ijk}\log\left(\frac{T_{ijk}B..^{2}}{T_{\ldots }B_{ik}B_{jk}}\right),$$ (Eq 8)

where *T*_*ijk*_ is the flow from node *i* to node *j* of element *k*, and *T*_…_ the total flow of all elements through all compartments [70]. The sensitivity of the compartment to its turnover time of a particular element is calculated by the partial derivative of *A*_*B*_ with respect to the biomass of element *k* in a particular compartment *p*:

$$\frac{\partial A_{B}}{\partial B_{pk}}=2\left(\frac{T\ldots }{B..}-\frac{1}{2}\frac{T_{.pk}+T_{p.k}}{B_{pk}}\right)$$ (Eq 9)

The amount of change in the ascendency value resulting from changes in turnover rate for each element (*k*) in each compartment (*p*) is interpreted as follows: a comparatively slow turnover rate results in a high sensitivity value, and this denotes the element that enters the node in the least relative proportion as defined by the consumer biomass stoichiometry (the limiting nutrient), and reiterates the results of a classical Liebig test. Negative sensitivities may arise for compartments when the turnover rate of a specific element is higher compared to the turnover rate of the entire system.

The identification of the limiting source of the limiting element is an extension of the classical Liebig notion. The sensitivities attributed to the individual flows from source *r* to sink *p* can be calculated as follows [70]:

$$\frac{\partial A_{B}}{\partial T_{rp}}=\log\left(\frac{T_{rp}B^{2}}{T_{..}B_{r}B_{p}}\right)$$ (Eq 10)

The limiting flow that depletes its source at the fastest rate compared to its availability results in the highest sensitivity value [70].

## Results

**Fig 2:**
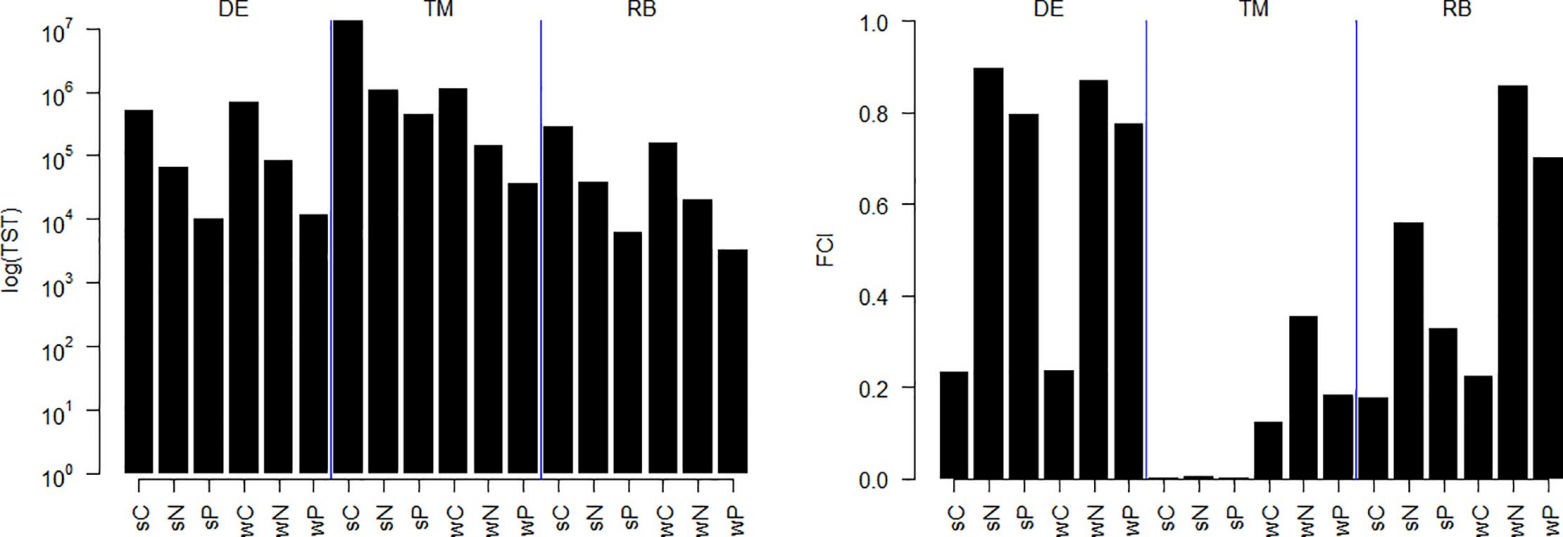
Total system Throughflow (TST) and Finn Cycling Index (FCI) of each network. DE = Durban Eddy, TM = Thukela Mouth, RB = Richards Bay, s = summer, w = winter. C = carbon, N = nitrogen, P = phosphorus.

The analysis of the ecosystem networks of the three different sub-regions of the KZN Bight revealed several differences. The largest subsystem, in terms of energy throughput was at the Thukela Mouth, followed by Durban Eddy and Richards Bay (Fig 2). As expected, the overall amount of carbon (C) flows was larger than that of nitrogen (N) and especially phosphorus (P). The Finn Cycling Index showed almost the opposite pattern–subsystems with highest throughflow had a lower degree of cycling. It was lowest in the TM region, especially in summer when more nutrients are input into this subsystem. The fraction cycled through the system was lowest for C, highest for N and intermediate for P in all three subsystems and both seasons (Fig 2).

**Fig 3:**
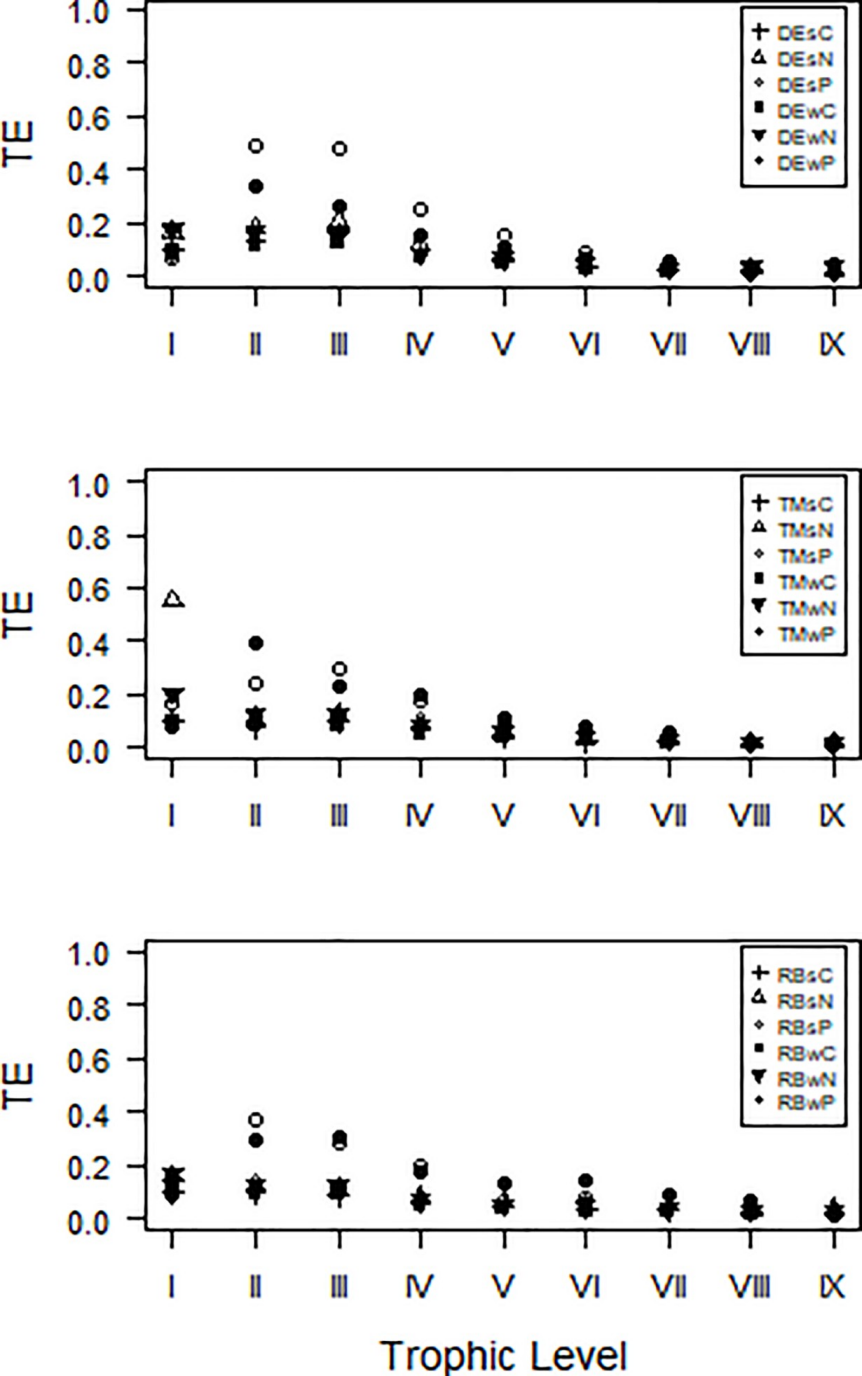
Trophic transfer efficiencies (as fraction) between discrete trophic levels of each nutrient (C, N, P) in each region (DE, TM, RB) and season (s, w).

Trophic Transfer Efficiencies (TE) generally decreased from lower to higher trophic levels (TL). At the DE in the southern Bight, TE were highest at TL II and III for all three nutrients and both seasons (Fig 3). This same pattern is apparent in the middle (TM) and northern (RB) Bight for P.

### Nutrient limitations

**Fig 4:**
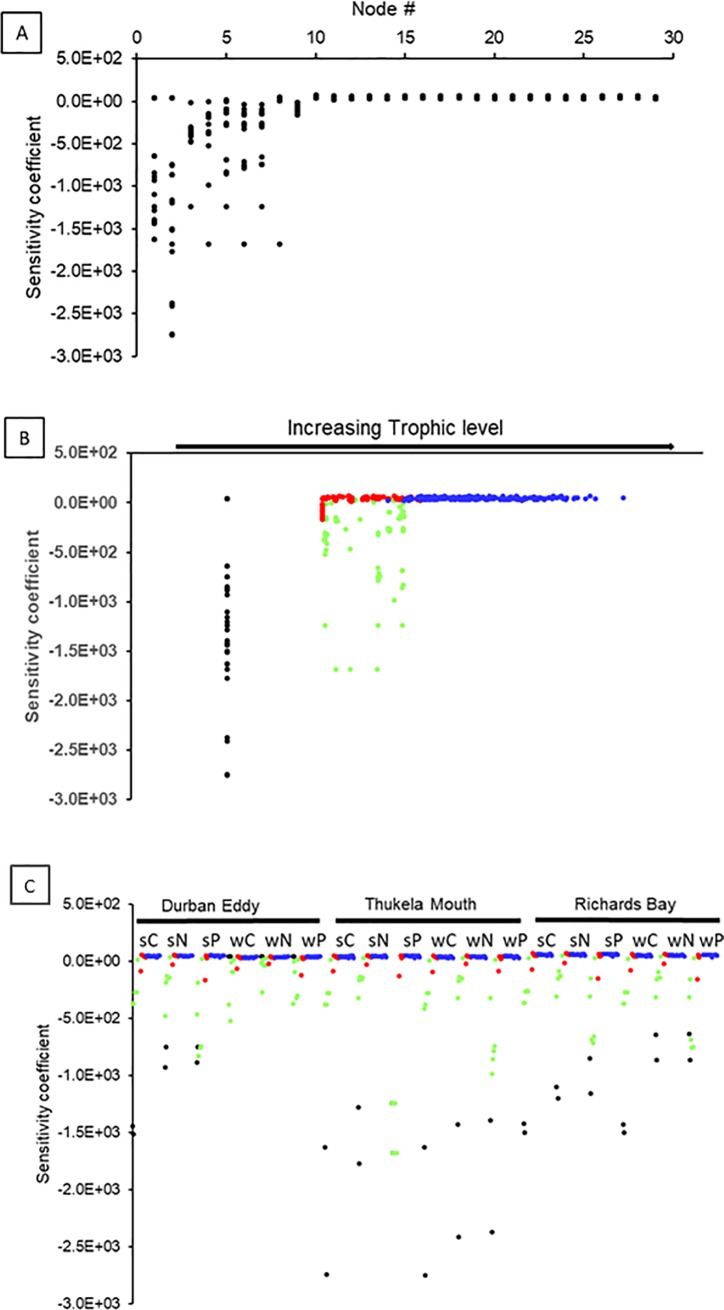
Node sensitivities for all 18 networks to changes to carbon, nitrogen and phosphorus turnover rates in each network calculated using Eq 9. A: Sensitivity values against number of compartments. B: sensitivity value against TL of compartments. C: Sensitivity value against TL of compartments for each region and season separately. Black: primary producers, Green: bacteria and zooplankton, Red: zoobenthos, Blue: fish and invertebrate nekton.

The sensitivity values describe the sensitivity of the ascendency to changes in biomass (C, N, P) of individual nodes. Nodes with higher turnover rate (T/B) compared to the system’s turnover rate have a negative sensitivity value, whereas nodes with a lower turnover rate contribute positively to ascendency. A similar pattern is apparent from all 18 networks, in that small organisms with fast turnover times show highly negative values, whereas the remainder of the nodes have positive values. Nutrient nodes are excluded from this analysis, as they do not have a nutrient ‘demand’, and thus no limitation in the sense of Liebig. In our example, nodes 1–7 and 9 have low sensitivity values throughout the bight and in both seasons (Fig 4). These nodes constitute groups of small organisms (Phytoplankton, Bacteria, Small Zooplankton, Small Macrobenthos). On the other hand, the nodes with a positive contribution to the system’s ascendency are larger sized organisms [various invertebrate and vertebrate groups] of nodes 8, 10 and above (Fig 4, S2 Table). These nodes have in addition very similar sensitivity values amongst each other, unlike the nodes representing smaller sized organisms, and for the three nutrients.

The node sensitivities for all three subsystems in the KZN Bight showed that P was the limiting nutrient to more than 50% of nodes (S6 Table). Around 40% of nodes were N limited, whereas C limitation occurred only in the Durban Eddy for Bacteria and Large Sharks. Diatoms and Flagellates were P limited in all areas and both seasons, sometimes joined by Bacteria and Heterotrophic Microplankton. Zooplankton and the benthos were mainly N limited, whereas most fish were P limited. Co-limitations, denoted by similar sensitivity values were apparent for all larger sized organisms [Nodes 8, 10 and above]. No distinct seasonal differences were apparent, with the exception of primary producer, small zooplankton and small macrobenthos nodes.

One striking difference between the three regions of the KZN Bight were the smaller sensitivity values (i.e. higher turnover rates) for diatoms and flagellates at TM, implying that nutrients were overall less limiting. This is in accordance with the comparatively high rate of nutrient delivery to this region from the Thukela River. In addition, the throughflow rate is higher in this area compared to DE and RB. Another difference of the TM region was the comparatively higher proportion of N, and thus lower P limitation, especially in summer (Table 2).

**Table 2:** Percentage of nodes limited by carbon (C), nitrogen (N) and phosphorus (P). DE: Durban Eddy, TM: Thukela Mouth, RB: Richard Bay, s: summer, w: winter.

	C	N	P
DEs	7.1	35.7	57.1
DEw	3.6	39.3	57.1
TMs	0	44.8	51.7
TMw	0	41.4	55.2
RBs	0	37.9	58.6
RBw	0	41.4	55.2

We compared the nodal nutrient limitations derived from the ascendency analysis (sensitivity values) to a more traditional approach of finding the difference between the stoichiometry of supply (consumption) and that of requirement (biomass). Both approaches yielded exactly the same results (S6 Table).

**Fig 5:**
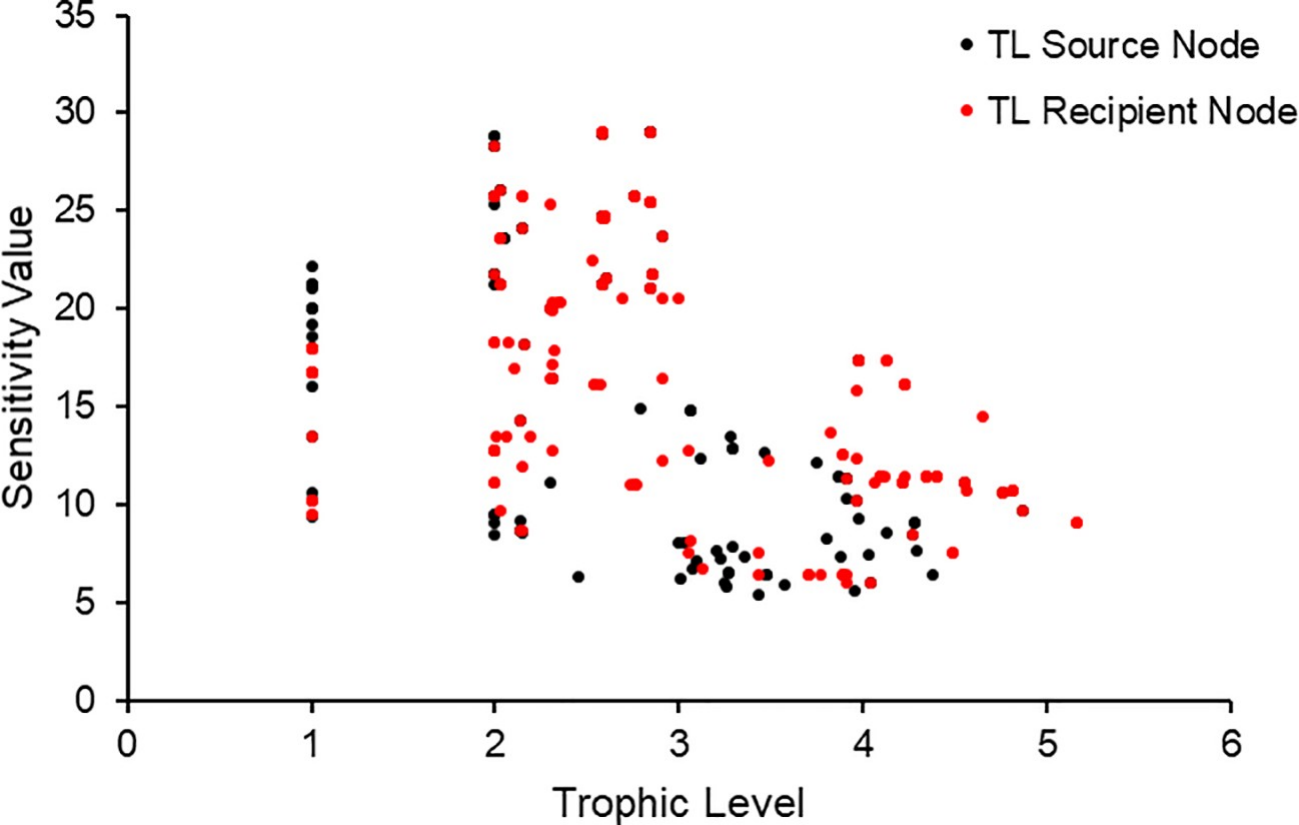
Sensitivity values of flows (calculated using Eq 10) are plotted against the effective trophic level of both the source and recipient compartment.

In contrast to the nutrient limitation of nodes, all flows in all areas and both seasons were limited through P, except for one occasion of an N limitation. This means that flow pathways for P deplete their sources the fastest of all three nutrients, and the demands of recipient nodes are higher compared to availability at the respective source node. Among the top five source nodes of the limiting flows for all regions and seasons were Diatoms, Flagellates, Small and Medium Copepods, for between 1 and 4 flows per network. Among the top five recipient nodes of limiting flows were Cetaceans in the TM and RB networks, whereas Small and Medium Copepods as well as Small Macrobenthos were either the source or recipients of limiting flows. In DE, limiting flows were sourced by a higher variety of nodes than in TM or RB. Neither the number of limiting flows nor the sensitivity values showed a significant relationship with the effective trophic level of either the source or the recipient node. However, higher trophic level nodes had overall lower sensitivity values, and thus deplete their source slower compared to lower trophic level nodes (Fig 5).

## Discussion

The analysis of multitrophic, stoichiometric networks of the KZN Bight expanded on previous studies in the region that investigated how subsystems are influenced by riverine input and oceanographic features such as upwelling [72–74]. A major influence of riverine input [including that from the Thukela River] was previously detected through nutrient supplements reaching the nearshore [41,56,75], and energy requirements of its food web [44]. Increased macrobenthos abundance and a unique demersal fish community diversity was found in the middle region of the bight at TM [46,76]. Increased bacteria standing stock but lower primary production were associated with allochthonous inputs from rivers [53,77], and high zooplankton biomass was associated with inshore regions of the bight and the St Lucia upwelling cell [78].

Our focus was on the functional attributes of the three subsystems, and on how stoichiometric constraints on a node level structure flows through ecosystems. The ecosystem attributes reported on here emanated from how the system deals with differences in stoichiometric demands and supplies of the various nodes. In addition to flows around individual nodes and between pairs of nodes, an important factor within the supply and demand chain is the speed with which nodes are re-supplied by recycling flows which themselves are subject to stoichiometric constraints by the nodes involved. These aspects influence the stoichiometry of supplies to nodes, and therefore play a role in the structure (e.g. predator-prey pairs, facultative feeding) and dynamics (e.g. nutrient turnover times) of ecosystems [79]. Firstly, the throughflow amount of C, N and P differed between the three subregions and nutrients, and was higher in summer compared to winter in the middle (TM) and northern bight (RB). These subregions experience inflow from large rivers, increased during the rainy season in spring and summer from the Thukela and Mfolozi Rivers respectively. RB was in addition influenced by the St Lucia upwelling, as also apparent from e.g. the high zooplankton biomass in the region [78]. The residence time of nutrients can be relatively high in the nearshore region of the middle to northern Bight, due to inshore currents flowing counter to the Agulhas current, and a swirl present at times in the mid-bight [73,80]. A longer residence time provides conditions to better utilise imported nutrients for primary production and by bacteria, and pass them on to higher trophic levels. The TEs were lower in the TM region, as more of the macronutrients were available compared to their demand. Although ecosystem productivity was higher in the TM region (see the TSTf, Fig 1), the primary productivity can be hindered by sediment loads from rivers which decrease light availability [53,81]. In all sub-regions, Transfer Efficiencies for P were higher compared to N, whereas the extent of cycling was higher for N. This might simply be a reflection of a more efficient recycling of N compared to P, and relatively more P being locked into biomass for a longer duration.

To our knowledge, only three other ecosystems have been presented as multitrophic networks of three macronutrients simultaneously. These are the Sylt Rømø Bight in the German/Danish Wadden Sea [82], Chesapeake Bay on the east coast of the USA [39], and an oligotrophic mangrove system offshore Belize [20]. The latter preserves the stoichiometry of nodes and flows throughout network construction and mass balancing, whereas the former two studies have constructed networks for the three nutrients in parallel. Similar to Chesapeake Bay, most nodes were P limited in our systems, whereas limitation results are not reported for the Sylt Rømø Bight. The latter exhibits comparable patterns to the KZN-Bight subsystems in transfer efficiencies (highest for P, lowest for C) and total material flow (lowest for P, highest for C). There was a difference in the amount of cycling, which was highest for P in the Sylt Rømø Bight, but highest for N in the KZN Bight. Our study area therefore experienced a different supply to demand ratio and a more efficient recycling of N relative to P.

### Nutrient limitations

The nutrient limitations of individual nodes were calculated from the stoichiometry of their biomass, and that of in- and outgoing links. The sensitivity values translate into differentiated nutrient limitations, as nodes with slower turnover rates for a particular nutrient have longer turnover times for that nutrient. The nutrient being available in the least relative proportion to the node can thus create a discrepancy between the stoichiometry of the biomass (C:N:P) and the turnover rates (T/B) of the elements within that stoichiometric constraint.

The limitations of individual nodes are indicated by sensitivity values that essentially describe the turnover time of nutrients within a node in comparison to that of the entire system. Slower turnover times compared to that of the system result in higher sensitivity values, which thus increased from smaller to larger organisms (Fig 4A), and from lower to higher trophic levels (Fig 4B) for all three nutrients (C, N, P). More than 50% of nodes were limited by P, which is in accordance with the higher transfer efficiencies for P compared to N and C. C limitation was apparent only from the southern bight (DE) to a small proportion.

It appears that the turnover rate of the three subsystems is held in balance by both smaller sized organisms with faster turnover rate than the system, and larger organisms with slower turnover rates. The difference in turnover rates between smaller and larger sized organisms is of course well documented in the literature for r and k strategists [83] and thus reinforces that the developmental state of ecosystems can be manifested in contrasting roles of their parts. Indeed, the contribution by both types of organisms in a network setting has been documented for their contribution to information [84,85]. Moreover, flow efficiency and redundant pathways as antagonistic agents existing in balance to increase the robustness of trophic networks have been documented previously as intrinsic features of such networks [86].

Higher trophic level (TL) organisms were less sensitive to different nutrient environments, in terms of limitations, compared to lower TL organism, expressed by more similar sensitivity values of the former. Lower TL organisms thus take a more prominent part in regulating different levels of nutrient supply for higher trophic levels. If higher TL organisms are less flexible to cope with variable stoichiometries [e.g. 87], lower TL organisms might afford their existence in a more stable stoichiometric environment and so facilitate ecosystem succession.

### Network structure, data, and considerations for future stoichiometry studies

Networks for all three KZN Bight subsystems were aggregated into the same number of groups, except where the absence or presence of species demanded otherwise. This choice was made to compare functional attributes (cycling, transfer efficiency, nutrient limitations) across different ecosystems, the calculation of which is influenced by the number of nodes and flows in a network [37,47,88]. The various networks in our study are as realistic a reflection of the subsystems as possible, and the equivalent aggregation facilitated a focus on the nutrient limitations by eliminating other, confounding factors attributable to aggregation.

The calculation of system-level attributes from the information embedded in nodes [species/species groups] and their interlinkages provided an opportunity to ascertain the influence of one level of hierarchy on another. Such an approach can be implemented in the Biodiversity-Ecosystem-Function (BEF) debate [89], as the sensitivity of ecological network analysis (ENA) metrics describing functional ecosystem properties to differences in number of nodes and flow magnitudes is well known, and used especially for linking these two hierarchies [node, ecosystem] [90,91][88,89]. However, a more detailed focus on biodiversity requires a higher detail of species-specific data.

Previous studies on stoichiometry and food webs have mainly been conducted for freshwater, rather than marine ecosystems. Experiments provide the bulk of data to freshwater studies [92], whereas in our data-based approach we constructed food webs based on biomass stoichiometry, and preserved this stoichiometry for resource-consumer flows, which were deducted from measurements or literature data. The former approach provides more exact data on the transformation of the stoichiometry during feeding interactions, whereas the latter approach facilitates a system’s view (e.g. system-wide recycling and transfer efficiencies) and allows to calculate the value of emerging properties of the food webs (e.g. system-wide nutrient limitations). Taking into account elemental content, nutrient ratios and fluxes has previously been mentioned as an important step in arriving at a comprehensive ecosystem view [93]. Merging both experimental and ecosystem analysis approaches could refine stoichiometric food webs by providing better constraints on recourse-consumer flow exchanges between living nodes, and with nutrient pools in multitrophic networks.

## Conclusions

The three subsystems of the oligo- to mesotrophic KwaZulu-Natal Bight were influenced by terrestrial and oceanic influences, and the former provided a distinct signal apparent in functional ecosystem attributes. By tracking the C:N:P stoichiometry, we found that all three subsystems were mainly limited by phosphorus, although in the middle bight the N limitation was comparatively higher at the expense of P limitation. Transfer efficiencies between trophic levels were lower in the TM region, indicating a higher availability of the three nutrients compared to their demand over all trophic levels, with an exception for N and P in winter. An interesting pattern was apparent for the range of sensitivity values of lower and higher trophic levels, indicating a divide between two groups of organisms: those with a faster turnover time than the system, and those with a slower turnover time. Such antagonistic behaviour has previously been detected for information related indices and might indicate a general feature of ecosystems. Lastly, to investigate the stoichiometry of ecosystems more comprehensively and better constrain nutrient flow data in ecosystem models, a merger of various established methodologies currently investigating ecological stoichiometry (experiments, metabolic constraints, ecosystem networks) can be useful.

## Supporting information

S1 Table Demersal functional groups and representative species of each sub-region where known. (DOCX)

S2 Table Input data for Ecopath Carbon networks of the DE, TM and RB susbsystems. P/B = Production/ Biomass, Q/B = Consumption/Biomass, EE = Ecotrophic Efficiency. EE and flows to detritus are proportions.
(DOCX)

S3 Table Diet compositions used in initial unbalanced Ecopath carbon networks. Group numbers refer to those in Table 4.1. Rows represent prey and columns represent predators. Groups 1 and 2 refer to primary producers and therefore do not require a predator column. I = imports. The first value in the column for group 28 was used in the DE networks and the second value was used in the TM and RB networks.
(DOCX)

S4 Table C:N and C:P ratios used to construct nitrogen and phosphorus networks. DE = Durban Eddy, TM = Thukela Mouth, RB = Richards Bay.
(DOCX)

S5 Table Carbon, nitrogen and phosphorus biomasses (g m-2) of groups in each network. Bold values represent biomasses estimated by Ecopath.
(DOCX)

S6 Table Nutrient limitations calculated from ascendency analysis, and turnover times in a node. DE: Durban Eddy, TM: Thukela Mouth, RB: Richard Bay. S: summer, w: winter. LN: Limiting Nutrient. C: carbon, N: nitrogen, P: phosphorus.
(DOCX)
