# Supplementary material for: Stoichiometric multitrophic networks reveal significance of land-sea interaction to ecosystem function in a subtropical nutrient-poor bight, South Africa
Source: PLoS One. 2019 Jan 7;14(1):e0210295. doi: 10.1371/journal.pone.0210295 (PMC6322777; doi:10.1371/journal.pone.0210295)
Supplement: S1 Table — (DOCX) [file pone.0210295.s001.docx]

S1:

| Functional group | Durban Eddy | Thukela Mouth | Richard Bay |
| --- | --- | --- | --- |
| Large suspension feeding macrobenthos | *Porifera spp.* | *Crinoid spp.* | *Porifera spp.* |
| Echinoderms | *Phormosoma spp.* | *Echinoidea spp.* | *Asteroidea spp.* |
| Molluscs (non-cephalopod) | *Phalium spp.* | *Sphenopus marsupialis* | *Phalium glaucum* |
| Prawns and shrimp | *Aristeomorpha foliacea* | *Harpiosquilla harpax* | *Penaeus japonicus* |
| Large Crustaceans | *Scyllarides elizabethae, Munida incerta* | *Parthenope squemvis, Scyramathia spp.* | *Portunus sanguinolinta* |
| Cuttlefish | *Sepia inserta, Sepia acuminata* | *Sepia vermicularis* | *Sepia inserta, Sepia acuminata* |
| Other cephalopods | *Ornithoteuthis volatilus, Notodarus hawaiiensis* | *Cephalopoda spp.* | *Cephalopoda spp.* |
| Flatfish | *Pseudorhombus spp., Citharoides macrolepis* | *Pseudorhombus spp.* | *Pseudorhombus elevatus* |
| Gurnards | *Chelidonichthys quecketti, Satyrichthys adeni* | *Lepidotrigla faurei* | *Lepidotrigla faurei* |
| Lizardfish | *Saurida undosquamis* | *Saurida undosquamis* | *Saurida undosquamis* |
| Other benthic carnivores | *Halieutaea fitzsimonsi, Hoplichthys acanthopleurus* | *Minous coccineus, Serranus knysnaensis* | *Cociella hemstraii* |
| Pinky | *Pomadasys olivaceum* | *Pomadasys olivaceum* | *Pomadasys olivaceum* |
| Red tjor-tjor | *Pagellus natalensis* | *Pagellus natalensis* | *Pagellus natalensis* |
| Other benthopelagic fish | *Polysteganus coeruleopunctatus, Neoscombrops annectens* | *Atrobucca nibe, Otolithes ruber* | *Upeneus vittatus, Lagocephalus guentheri* |
| Skates and rays | *Rhinobatus holcorhynchos* | *Raja miraletus, Dasyatis chrysonata* | not caught |
| Small benthic sharks | *Mustelus mosis, Pliotrema warreni* | *Halaelurus lineatus* | not caught |
