# Supplementary material for: Stoichiometric multitrophic networks reveal significance of land-sea interaction to ecosystem function in a subtropical nutrient-poor bight, South Africa
Source: PLoS One. 2019 Jan 7;14(1):e0210295. doi: 10.1371/journal.pone.0210295 (PMC6322777; doi:10.1371/journal.pone.0210295)
Supplement: S2 Table — P/B = Production/ Biomass, Q/B = Consumption/Biomass, EE = Ecotrophic Efficiency. EE and flows to detritus are proportions. (DOCX) [file pone.0210295.s002.docx]

S2:

|  |  |  |  |  | Flows to detritus | | |
| --- | --- | --- | --- | --- | --- | --- | --- |
| Functional Groups/Species | Biomass  reference | P/B(y^-1^) | Q/B(y^-1^) | EE | Suspended  POC | Sediment POC | DOC |
| 1 Diatoms | (1) | 199-490* | n/a |  | 1.00 |  |  |
| 2 Flagellates | “ | 208-410* | n/a |  | 1.00 |  |  |
| 3 Bacteria | (2) | 119.00^a^ | 215.00^p^ | 0.95^aa^ | 1.00 |  |  |
| 4 Heterotrophic microplankton | (3) | 100.00^b^ | 215.00^p^ | 0.95^aa^ | 1.00 |  |  |
| 5 Small copepods | (4) | 40.00^c^ | 165.00^c^ |  | 1.00 |  |  |
| 6 Medium copepods | “ | 40.00^c^ | 165.00^c^ |  | 1.00 |  |  |
| 7 Large copepods | “ | 40.00^c^ | 165.00^c^ |  | 1.00 |  |  |
| 8 Other large zooplankton | “ | 8.70^d^ | 29.00^d^ |  | 1.00 |  |  |
| 9 Small macrobenthos | Estimated by  Ecopath | 6.32^e^ | 70.20^e^ | 0.95^aa^ |  | 1.00 |  |
| 10 Large suspension feeders |  | 0.25^f^ | 0.50^f^ | 0.95^aa^ |  | 1.00 |  |
| 11 Echinoderm | Estimated by Ecopath, or (5) | 1.20^g^ | 4.00^g^ | 0.95^aa^ |  | 1.00 |  |
| 12 Molluscs (non-cephalopod) | Estimated by Ecopath, or (5) | 2.50^g^ | 8.20^g^ | 0.95^aa^ |  | 1.00 |  |
| 13 Prawn and shrimp | Estimated by Ecopath, or (5) | 2.50-2.73* | 6.10^q^ | 0.95^aa^ |  | 1.00 |  |
| 14 Large crustaceans | (5), collected in conjunction with (6) | 1.60^h^ | 10.00^h^ |  |  | 1.00 |  |
| 15 Cuttlefish | Estimated by  Ecopath | 1.10^g^ | 3.50^g^ | 0.95^aa^ |  | 1.00 |  |
| 16 Other cephalopods |  | 1.95^i^ | 3.90^i^ | 0.95^aa^ |  | 1.00 |  |
| 17 Flatfish | (5), collected in conjunction with (6),  or estimated by Ecopath  “  “  “  “ | 1.17^g^ | 6.80^r^ |  |  | 1.00 |  |
| 18 Gurnard |  | 0.57^j^ | 3.50^s^ |  |  | 1.00 |  |
| 19 Lizardfish |  | 2.13^k^ | 6.20^t^ |  |  | 1.00 |  |
| 20 Other benthic carnivorous fish |  | 2.13^k^ | 7.30^v^ |  |  | 1.00 |  |
| 21 Red tjor-tjor |  | 2.70^l^ | 6.90^t^ |  |  | 1.00 |  |
| 22 Pinky |  | 0.65^d^ | 4.80^t^ |  |  | 1.00 |  |
| 23 Other benthopelagic fish |  | 2.31^k^ | 6.20^v^ |  |  | 1.00 |  |
| 24 Small pelagic fish | Estimated by  Ecopath | 2.00^m^ | 11.20^w^ | 0.95^aa^ |  | 1.00 |  |
| 25 Large pelagic fish |  | 0.87^n^ | 8.98^x^ | 0.43^ab^ |  | 1.00 |  |
| 26 Skates and rays | (5), collected in conjunction with (6),  or estimated by Ecopath | 0.92^g^ | 2.60^t^ | 0.66^g^ |  | 1.00 |  |
| 27 Small benthic sharks |  | 0.50^g^ | 3.10^y^ |  |  | 1.00 |  |
| 28 Large sharks | Estimated by Ecopath | 0.10^o^ | 1.75^z^ | 0.10^ac^ |  | 1.00 |  |
| 29 Cetaceans | Estimated by Ecopath | 6.00^c^ | 10.00^c^ |  |  | 1.00 |  |
| 30 Suspended POM | (7) | n/a | n/a | n/a |  | 0.50 | 0.50 |
| 31 Sediment POM | (8) | n/a | n/a | n/a |  |  | 1.00 |
| 32 DOM | (5) | n/a | n/a | n/a |  |  | 1.00 |

References: *see text; a: (2); b: (9); c: (10); d: (11); e: (12); f: “bottom living structures” in (13); g: (14); h: (15); i: “squid” in (16); j: (17); k: (18); l: (19); m: (20); n: (21); o: intrinsic rate of increase of *S. lewini* from (22); p: (9); q: (23); r: average of *Citharoides macrolepis* and *Pseudorhombus natalensis* calculated using Fishbase (24); s: average of *Satirichthys adeni* and *Chelidonithys quecketti* calculated using Fishbase (24); t: (24); u: average of *Hoplichthys acanthopleurus* and *Halieutaea fitzsimonsi* calculated using Fishbase (24); v: average of *Neoscombrops annectens*, *Histiopterus typus* and *Plysteganus coeruleopunctatus* calculated using Fishbase (24); w: (25); x: average of *Scomberomorus commerson*, *Thunnus albacares*, *Coryphaena hippurus*, *Euthynnus affinis* in Fishbase (24); y: average of *Squalus megalops* and *Pliotrema warren* using Fishbase (24); z: average of *Sphyrna mokorran*, *Sphyrna lewini* and *Isurus oxyrinchus* using Fishbase (24); aa: (26); ab: (27); ac: (28).

References to S2 Table:

1. Barlow RG, Lamont T, Gibberd MJ, van den Berg M, Britz K. Chemotaxonomic investigation of phytoplankton in the shelf ecosystem of the KwaZulu-Natal Bight, South Africa. African J Mar Sci. 2015;37(4):467–84.

2. Muir D, Kunnen T, Scharler U. A seasonal comparison of prokaryote numbers, biomass and heterotrophic productivity in waters of the KwaZulu-Natal Bight, South Africa. African J Mar Sci [Internet]. 2016;38(sup1):S123–38. Available from: http://www.tandfonline.com/doi/full/10.2989/1814232X.2016.1146630

3. Moyo R. Carbon biomass and processing by protists in the Natal Bight. University of KwaZulu-Natal, Durban, South Africa; 2011.

4. Pretorius M, Huggett JA, Gibbons MJ. Summer and winter differences in zooplankton biomass, distribution and size composition in the KwaZulu-Natal Bight, South Africa. African J Mar Sci. 2016;38:S155–68.

5. Ayers MJ, Scharler UM, Fennessy ST. Modelling ecosystem effects of reduced prawn recruitment on the Thukela Bank trawling grounds, South Africa, following nursery loss. Mar Ecol Prog Ser [Internet]. 2013 Apr 8 [cited 2014 Jun 4];479:143–61. Available from: http://www.int-res.com/abstracts/meps/v479/p143-161/

6. Fennessy ST. Subtropical demersal fish communities on soft sediments in the KwaZulu-Natal Bight, South Africa. African J Mar Sci. 2016;38:S169–80.

7. Omarjee A. Phytoplankton Studies in the KwaZulu-Natal Bight. 2013.

8. Scharler UM, Ayers MJ, de Lecea AM, Fennessy ST, Pretorious M, Huggett JA, et al. Riverine influence determines nearshore heterogeneity of nutrient (C,N,P) content and stoichiometry in the KwaZulu-Natal Bight, South Africa. African J Mar Sci. 2016;38(Supplement):S193–4.

9. Opitz S. Trophic interactions in Caribbean coral reefs. Manila, Philippines; 1996.

10. Toral-Granda M., Moloney CL, Harris JM, Mann BQ. Ecosystem Impacts of the KwaZulu-Natal Reef Fishery, South Africa: An Exploratory Model. In: Ecosystem Approaches for Fisheries Management [Internet]. Alaska Sea Grant College Program, AK-SG-99-01; 1999. p. 211–30. Available from: http://seagrant.uaf.edu/bookstore/pubs/AK-SG-99-01.html

11. Okey TA, Vargo GA, MacKinson S, Vasconcellos M, Mahmoudi B, Meyer CA. Simulating community effects of sea floor shading by plankton blooms over the West Florida Shelf. Ecol Modell. 2004;172(2–4):339–59.

12. Rocha GRA, Rossi-Wongtschowski CLDB, Pires-Vanin AMS, Soares LSH. Trophic models of São Sebastião Channel and continental shelf systems, SE Brazil. Panam J Aquat Sci. 2007;2(2):149–62.

13. Cheung W-L, Watson R, Pitcher TJ. Policy simulation of fisheries in the Hong Kong marine ecosystem [Internet]. Vol. 10, The use of ecosystem models to investigate multispecies management strategies for capture fisheries. 2002 Sep. Available from: http://linkinghub.elsevier.com/retrieve/pii/S0735109710019121

14. Amorim P, Duarte G, Guerra M, Morato T, Stobberup K. Preliminary Ecopath model of the Guinea-Bissau continental shelf ecosystem (NW-Africa). In: Palomares M, Pauly D, editors. West African coatal ecosystems. Fisheries Centre Research Reports, Fisheries Centre, UBC, Vancouver; 2004. p. 95–112.

15. Morato T, Pitcher TJ. Ecosystem Simulations of Management Strategies for Data- Limited Seamount Fisheries. In: Fisheries Assessment and Management in Data-Limited Situations. 2005. p. 467–86.

16. Gasalla M, Rossi-Wongtschowski CLDB. Contribution of ecosystem analysis to investigating the effects of changes in fishing strategies in the South Brazil Bight coastal ecosystem. Ecol Modell [Internet]. 2004 Mar 1 [cited 2011 Jun 14];172(2–4):283–306. Available from: http://linkinghub.elsevier.com/retrieve/pii/S0304380003003740

17. Stanford RJ, Pitcher TJ. Ecosystem Simulations of the English Channel: Climate and Trade-Offs [Internet]. Vol. 12, Fisheries Centre Research Reports. 2004. Available from: http://www.fisheries.ubc.ca/publications/ecosystem-simulations-english-channel-climate-and-trade-offs

18. Duan LJ, Li SY, Liu Y, Jiang T, Failler P. A trophic model of the Pearl River Delta coastal ecosystem. Ocean Coast Manag [Internet]. 2009;52(7):359–67. Available from: http://dx.doi.org/10.1016/j.ocecoaman.2009.04.005

19. Angelini R, Vaz-Velho F. Ecosystem structure and trophic analysis of Angolan fishery landings. Sci Mar [Internet]. 2010;75(2):309–19. Available from: http://scientiamarina.revistas.csic.es/index.php/scientiamarina/article/view/1254/1324

20. de Paula E Silva R, Sousa MI, Caramelo AM. The Maputo Bay ecosystem (Mozambique). In: Christensen V, Pauly D, editors. Trophic models of aquatic ecosystems ICLARM Conference Proceedings. 1993. p. 214–23.

21. Govender A. Mortality and biological reference points for the king mackerel (Scomberomorus commerson) fishery off Natal, South Africa (based on a per-recruit assessment). Fish Res. 1995;23(3–4):195–208.

22. Dudley SFJ, Simpfendorfer C a. Population status of 14 shark species caught in the protective gillnets off KwaZulu–Natal beaches, South Africa, 1978–2003. Mar Freshw Res [Internet]. 2006;57(2):225. Available from: http://www.publish.csiro.au/?paper=MF05156

23. Maynou F, Cartes J. Field estimation of daily ration in deep-sea shrimp Aristeus antennatus (Crustacea: Decapoda) in the western Mediterranean. Mar Ecol Prog Ser. 1997;153:191–6.

24. Froese R, Pauly D. World Wide Web electronic publication. www.fishbase.org [Internet]. 2018. Available from: www.fishbase.org

25. Vasconcellos M. Ecosystem impacts of fishing forage fishes: an analysis of harvest strategies for the Brazilian sardine. University of British Columbia; 2000.

26. Christensen V, Walters C, Pauly D, Forrest R. Ecopath with Ecosim version 6. User Guide. 2008.

27. Freire KMF, Christensen V, Pauly D. Description of the East Brazil Large Marine Ecosystem using a trophic model. Sci Mar [Internet]. 2008;72(3):477–91. Available from: http://www.vliz.be/imisdocs/publications/140095.pdf

28. Ayers MJ, Scharler UM. Use of sensitivity and comparative analyses in constructing plausible trophic mass-balance models of a data-limited marine ecosystem - The KwaZulu-Natal Bight, South Africa. J Mar Syst. 2011;88(2):298–311.
