# Supplementary material for: Stoichiometric multitrophic networks reveal significance of land-sea interaction to ecosystem function in a subtropical nutrient-poor bight, South Africa
Source: PLoS One. 2019 Jan 7;14(1):e0210295. doi: 10.1371/journal.pone.0210295 (PMC6322777; doi:10.1371/journal.pone.0210295)
Supplement: S3 Table — Group numbers refer to those in Table 4.1. Rows represent prey and columns represent predators. Groups 1 and 2 refer to primary producers and therefore do not require a predator column. I = imports. The first value in the column for group 28 was used in the DE networks and the second value was used in the TM and RB networks. (DOCX) [file pone.0210295.s003.docx]

S3:

|  | 3 | 4 | 5 | 6 | 7 | 8 | 9 | 10 | 11 | 12 | 13 | 14 | 15 | 16 | 17 | 18 | 19 | 20 | 21 | 22 | 23 | 24 | 25 | 26 | 27 | 28 | 29 |
| --- | --- | --- | --- | --- | --- | --- | --- | --- | --- | --- | --- | --- | --- | --- | --- | --- | --- | --- | --- | --- | --- | --- | --- | --- | --- | --- | --- |
| 1 | 0.015 | 0.015 | 0.467 | 0.216 | 0.216 | 0.058 | 0.057 | 0.042 | 0.012 |  | 0.050 |  |  |  |  |  |  |  |  |  |  | 0.018 |  |  |  |  |  |
| 2 | 0.015 | 0.015 | 0.333 | 0.154 | 0.154 | 0.042 | 0.041 | 0.030 | 0.008 |  | 0.050 |  |  |  |  |  |  |  |  |  |  | 0.013 |  |  |  |  |  |
| 3 |  |  | 0.130 | 0.399 | 0.399 | 0.639 | 0.360 | 0.927 | 0.130 | 0.050 |  |  |  |  |  |  |  |  |  |  |  |  |  |  |  |  |  |
| 4 |  |  | 0.000 | 0.001 | 0.001 | 0.001 |  |  | 0.000 | 0.000 |  |  |  |  |  |  |  |  |  |  |  |  |  |  |  |  |  |
| 5 |  |  | 0.020 | 0.120 | 0.120 | 0.120 |  |  |  |  | 0.033 |  |  |  |  |  |  |  |  |  |  | 0.344 |  |  |  |  |  |
| 6 |  |  |  | 0.010 | 0.010 | 0.057 |  |  |  |  | 0.033 |  |  |  |  |  |  |  |  |  |  | 0.204 |  |  |  |  |  |
| 7 |  |  |  |  |  | 0.043 |  |  |  |  | 0.033 |  |  |  |  |  |  |  |  |  | 0.283 | 0.151 |  |  |  |  |  |
| 8 |  |  |  |  |  | 0.012 |  |  |  |  |  |  |  |  | 0.030 | 0.001 |  |  | 0.049 | 0.045 | 0.025 | 0.084 | 0.000 |  |  |  |  |
| 9 |  |  |  |  |  |  | 0.050 |  | 0.301 | 0.189 | 0.200 | 0.543 | 0.286 | 0.460 | 0.630 | 0.287 | 0.275 | 0.275 | 0.069 | 0.105 | 0.550 | 0.100 | 0.003 | 0.117 | 0.204 |  |  |
| 10 |  |  |  |  |  |  |  |  | 0.103 | 0.100 |  | 0.188 | 0.041 |  |  |  | 0.029 | 0.029 | 0.002 | 0.399 |  |  |  | 0.303 |  |  |  |
| 11 |  |  |  |  |  |  |  |  | 0.002 | 0.001 |  | 0.020 |  |  | 0.057 |  |  |  | 0.025 | 0.001 |  |  |  | 0.021 |  |  |  |
| 12 |  |  |  |  |  |  |  |  |  |  |  | 0.019 |  |  | 0.080 | 0.001 |  |  |  |  |  |  |  | 0.014 |  |  |  |
| 13 |  |  |  |  |  |  |  |  |  |  |  | 0.010 | 0.049 | 0.080 | 0.050 | 0.041 | 0.196 | 0.196 | 0.004 | 0.257 | 0.088 | 0.001 | 0.002 | 0.085 | 0.053 |  |  |
| 14 |  |  |  |  |  |  |  |  |  |  |  | 0.005 | 0.333 | 0.241 | 0.116 | 0.131 | 0.127 | 0.127 | 0.009 | 0.113 |  |  | 0.006 |  | 0.204 | 0.002, 0.002 |  |
| 15 |  |  |  |  |  |  |  |  |  |  |  |  | 0.091 |  |  | 0.007 | 0.039 | 0.039 |  | 0.068 |  |  | 0.010 | 0.028 | 0.016 | 0.011, 0.009 | 0.170 |
| 16 |  |  |  |  |  |  |  |  |  |  |  |  | 0.017 | 0.138 |  | 0.004 | 0.029 | 0.029 |  | 0.001 |  |  | 0.023 | 0.171 | 0.166 | 0.055, 0.028 | 0.075 |
| 17 |  |  |  |  |  |  |  |  |  |  |  |  |  |  | 0.010 | 0.401 | 0.059 | 0.059 |  |  |  |  |  | 0.118 |  | 0.012, 0.002 | 0.004 |
| 18 |  |  |  |  |  |  |  |  |  |  |  |  |  |  |  | 0.002 | 0.098 | 0.098 |  |  |  |  |  | 0.014 |  | 0.003, 0 |  |
| 19 |  |  |  |  |  |  |  |  |  |  |  |  |  |  |  |  |  |  |  |  |  |  |  |  |  | 0.007, 0.001 | 0.002 |
| 20 |  |  |  |  |  |  |  |  |  |  |  |  | 0.183 |  |  | 0.036 |  |  |  |  |  |  | 0.000 |  | 0.129 | 0.006, 0.025 | 0.043 |
| 21 |  |  |  |  |  |  |  |  |  |  |  |  |  |  |  |  |  |  |  |  |  |  | 0.042 |  |  | 0.005, 0.003 | 0.020 |
| 22 |  |  |  |  |  |  |  |  |  |  |  |  |  |  |  |  |  |  |  |  |  |  | 0.001 |  |  | 0.006, 0.005 | 0.060 |
| 23 |  |  |  |  |  |  |  |  |  |  |  |  |  |  | 0.010 |  | 0.147 | 0.147 | 0.842 | 0.011 |  |  | 0.085 | 0.052 | 0.085 | 0.175, 0.073 | 0.117 |
| 24 |  |  |  |  |  |  |  |  |  |  |  |  |  | 0.080 | 0.010 | 0.009 |  |  |  |  |  |  | 0.719 |  | 0.145 | 0.007, 0.162 | 0.428 |
| 25 |  |  |  |  |  |  |  |  |  |  |  |  |  |  |  |  |  |  |  |  |  |  | 0.101 |  |  | 0.0478, 0.056 | 0.054 |
| 26 |  |  |  |  |  |  |  |  |  |  |  |  |  |  |  | 0.081 |  |  |  |  |  |  |  | 0.019 |  | 0.275, 0.148 | 0.007 |
| 27 |  |  |  |  |  |  |  |  |  |  |  |  |  |  |  |  |  |  |  |  |  |  |  |  |  | 0.092, 0.068 |  |
| 28 |  |  |  |  |  |  |  |  |  |  |  |  |  |  |  |  |  |  |  |  |  |  |  |  |  | 0.271, 0.2 |  |
| 29 |  |  |  |  |  |  |  |  |  |  |  |  |  |  |  |  |  |  |  |  |  |  |  |  |  | 0, 0.076 |  |
| 30 | 0.040 | 0.040 | 0.050 | 0.040 | 0.040 | 0.028 | 0.033 |  | 0.123 | 0.300 |  |  |  |  |  |  |  |  |  |  |  | 0.076 |  |  |  |  |  |
| 31 | 0.010 | 0.010 |  | 0.060 | 0.060 |  | 0.458 |  | 0.320 | 0.360 | 0.600 | 0.215 |  |  | 0.007 |  |  |  |  |  | 0.055 | 0.010 |  | 0.057 |  |  |  |
| 32 | 0.920 | 0.920 |  |  |  |  |  |  |  |  |  |  |  |  |  |  |  |  |  |  |  |  |  |  |  |  |  |
| I |  |  |  |  |  |  |  |  |  |  |  |  |  |  |  |  |  |  |  |  |  |  | 0.008 |  |  | 00246, 0.135 | 0.020 |

Calculated using: 3-12: (1); 13: (2); 14: (3); 15: (4); 16: (5); 17: (6); 18: (7); 19, 20: (8); 21: (9); 22: (10); 23: (11); 24: (1); 25: (12); 26: (6); 27: (13); 28 (DE): (14), (15), (16); 28 (TM and RB): (17), (18), (19), (20), (21), (22), (23); 29: (24), (25).

References to S3 Table:

1. Okey TA, Vargo GA, MacKinson S, Vasconcellos M, Mahmoudi B, Meyer CA. Simulating community effects of sea floor shading by plankton blooms over the West Florida Shelf. Ecol Modell. 2004;172(2–4):339–59.

2. Rainer SF. Diet of prawns from the continental slope of north-western Australia. Bull Mar Sci. 1992;50(2):258–74.

3. Okey TA, Meyer C. An ecosystem model of the West Florida shelf for use in fisheries management and ecological research. Volume II. Model construction. St Petersburg; 2002.

4. Castro B, Guerra Á. The diet of Sepia officinalis (Linnaeus, 1758) and Sepia elegans (D’Orbigny, 1835) (Cephalopoda, Sepioidea) from teh Ría de Vigo (NW Spain). Sci Mar. 1990;54(4):375–88.

5. de Lecea AM, Smit AJ, Fennessy ST. Riverine dominance of a nearshore marine demersal food web: evidence from stable isotope and C/N ratio analysis. African J Mar Sci. 2016;38:S181–92.

6. Amorim P, Duarte G, Guerra M, Morato T, Stobberup K. Preliminary Ecopath model of the Guinea-Bissau continental shelf ecosystem (NW-Africa). In: Palomares M, Pauly D, editors. West African coatal ecosystems. Fisheries Centre Research Reports, Fisheries Centre, UBC, Vancouver; 2004. p. 95–112.

7. Meyer M, Smale MJ. Predation patterns of demersal teleosts from the Cape south and west coasts of South Africa. 1. Pelagic predators. South African J Mar Sci. 1991;10(1):173–91.

8. Duan LJ, Li SY, Liu Y, Jiang T, Failler P. A trophic model of the Pearl River Delta coastal ecosystem. Ocean Coast Manag [Internet]. 2009;52(7):359–67. Available from: http://dx.doi.org/10.1016/j.ocecoaman.2009.04.005

9. Morato T, Pitcher TJ. Ecosystem Simulations of Management Strategies for Data- Limited Seamount Fisheries. In: Fisheries Assessment and Management in Data-Limited Situations. 2005. p. 467–86.

10. Joubert CSW, Hanekom PB. A Study of Feeding in Some Inshore Reef Fish of the Natal Coast , South Africa A study of feeding in some inshore reef fish of the Natal Coast , South Africa. South African J Zool. 1980;15(4):262–74.

11. Hajisamae S. Estuarine , Coastal and Shelf Science Trophic ecology of bottom fishes assemblage along coastal areas of Thailand. Estuar Coast Shelf Sci [Internet]. 2009;82:503–14. Available from: http://dx.doi.org/10.1016/j.ecss.2009.02.010

12. Potier M, Marsac F, Cherel Y, Lucas V, Sabatié R, Maury O, et al. Forage fauna in the diet of three large pelagic fishes (lancetfish , swordfish and yellowfin tuna) in the western equatorial Indian Ocean. Fish Res. 2007;83:60–72.

13. Ebert DA, Compagno LJ V, Cowley PD. A preliminary investigation of the feeding ecology of squaloid sharks off the west coast of southern Africa. South African J Mar Sci. 1992;12(1):601–9.

14. Cliff G. Sharks caught in the protective gill nets off KwaZulu-Natal , South Africa. 8. The Great hammerhead shark Sphyrna mokarran (Rüppell). S Afr J Sci. 1995;15(1):105–14.

15. Cliff G, Dudley SFJ, Davis B. Sharks caught in the protective gill nets off Natal , South Africa. 3. The shortfin mako shark Isurus oxyrinchus (Rafinesque). South African J Mar Sci. 1990;9(1):115–26.

16. Bruyn P De, Dudley SFJ, Cliff G, Smale MJ. Sharks caught in the protective gill nets off KwaZulu-Natal , South Africa. 11. The scalloped hammerhead shark Sphyrna lewini (Griffith and Smith). African J Mar Sci. 2005;27(3):517–28.

17. Aitken A. The biology and tourism potential of the tiger shark Galocerdo cuvier and the whale shark Rhinocodon typus in KwaZulu-Natal, South Africa. University of Cape Town, South Africa; 2003.

18. Cliff G, Dudley SFJ, Davis B. Sharks caught in the protective gill nets off Natal , South Africa. 2. The great white shark Carcharodon carcharias (Linnaeus). South African J Mar Sci. 1989;8(1):131–44.

19. Cliff G, Dudley SFJ. Sharks caught in the protective gill nets off Natal , South Africa. 4. The bull shark Carcharhinus leucas Valenciennes. South African J Mar Sci. 1991;10(1):253–70.

20. Cliff G, Dudley SFJ. Sharks caught in the protective gill nets off Natal , South Africa. 5. The Java shark Carcharhinus amboinensis (Müller & Henle). South African J Mar Sci. 1991;11(1):443–53.

21. Allen BR, Cliff G. Sharks caught in the protective gill nets off Kwazulu-Natal , South Africa. 9. The spinner shark Carcharhinvs brevipinna (Müller and Henle). South African J Mar Sci. 2000;22(1):199–215.

22. Dudley SFJ, Cliff G. Sharks caught in the protective gill nets off Natal , South Africa. 7. The blacktip shark Carcharhinus limbatus (Valenciennes). South African J Mar Sci. 1993;13(1):237–54.

23. Dudley SFJ, Cliff G, Zungu MP, Smale MJ. Sharks caught in the protective gill nets off KwaZulu-Natal , South Africa. 10. The dusky shark Carcharhinus obscurus (Lesueur 1818). African J Mar Sci. 2005;27(1):107–27.

24. Young D, Cockroft V. Diet of common dolphins (Delphinus delphis) off the south-east coast of southern Africa: opportunism or specialisation? J Zool. 1994;234:41–35.

25. Cockcroft V, Ross G. Food and feeding of the Indian Ocean bottlenose dolphin off southern Natal, South Africa. In: Leatherwood S, Reeves R, editors. The bottlenose dolphin. San Diego: Academic Press; 1990.
