# Supplementary material for: Stoichiometric multitrophic networks reveal significance of land-sea interaction to ecosystem function in a subtropical nutrient-poor bight, South Africa
Source: PLoS One. 2019 Jan 7;14(1):e0210295. doi: 10.1371/journal.pone.0210295 (PMC6322777; doi:10.1371/journal.pone.0210295)
Supplement: S4 Table — DE = Durban Eddy, TM = Thukela Mouth, RB = Richards Bay. (DOCX) [file pone.0210295.s004.docx]

S4:

|  |  | C:N | | | C:P | | |
| --- | --- | --- | --- | --- | --- | --- | --- |
|  | Functional group/species | DE | TM | RB | DE | TM | RB |
| 1 | Diatoms | 8.20^a^ | 8.2^a^ | 8.2^a^ | 20.00^a^ | 20.00^a^ | 20.00^a^ |
| 2 | Flagellates | 8.20^a^ | 8.2^a^ | 8.2^a^ | 20.00^a^ | 20.00^a^ | 20.00^a^ |
| 3 | Bacteria | 7.80^a^ | 7.8^a^ | 7.8^a^ | 38.36^a^ | 38.36^a^ | 38.36^a^ |
| 4 | Heterotrophic microplankton | 5.31^b^ | 5.31^b^ | 5.31^b^ | 48.05^b^ | 48.05^b^ | 48.05^b^ |
| 5 | Small copepods | 5.14^c^ | 4.87^c^ | 5.49^c^ | 126.1^c^ | 138.19^c^ | 100.22^c^ |
| 6 | Medium copepods | 5.00^c^ | 4.95^c^ | 5.36^c^ | 264.24^c^ | 110.98^c^ | 140.52^c^ |
| 7 | Large copepods | 4.91^c^ | 4.94^c^ | 5.34^c^ | 242.98^c^ | 141.09^c^ | 109.62^c^ |
| 8 | Other large zooplankton | 3.62^d^ | 3.62^d^ | 3.62^d^ | 22.27^d^ | 22.27^d^ | 22.27^d^ |
| 9 | Small macrobenthos | 4.67^e^ | 4.67^e^ | 4.67^e^ | 87.00^k^ | 87.00^k^ | 87.00^k^ |
| 10 | Large suspension feeders | 4.99^c^ | 4.99^c^ | 4.99^c^ | 183.83^c^ | 183.83^c^ | 183.83^c^ |
| 11 | Echinoderms | 4.16 ^f^ | 4.16 ^f^ | 4.16 ^f^ | 182.00^f^ | 182.00^f^ | 182.00^f^ |
| 12 | Molluscs (non-cephalopod) | 4.19^c^ | 4.19^c^ | 4.19^c^ | 265.12^c^ | 265.12^c^ | 265.12^c^ |
| 13 | Prawn and shrimp | 3.85^c^ | 3.85^c^ | 3.85^c^ | 88.08^c^ | 88.08^c^ | 88.08^c^ |
| 14 | Large crustaceans | 3.82^c^ | 3.83^c^ | 4.03^c^ | 38.38^c^ | 25.92^c^ | 34.56^c^ |
| 15 | Cuttlefish | 4.00^c^ | 3.76^c^ | 3.76^c^ | 34.07^c^ | 31.92^c^ | 31.92^c^ |
| 16 | Other cephalopods | 3.90^c^ | 3.90^c^ | 3.90^c^ | 33.06^c^ | 33.06^c^ | 33.06^c^ |
| 17 | Flatfish | 3.80^c^ | 3.74^c^ | 3.73^c^ | 43.86^c^ | 51.01^c^ | 27.78^c^ |
| 18 | Gurnard | 3.81^c^ | 3.80^c^ | 3.87^c^ | 50.60^c^ | 295.76^c^ | 31.12^c^ |
| 19 | Lizardfish | 3.65^c^ | 3.72^c^ | 3.70^c^ | 38.54^c^ | 39.25^c^ | 33.99^c^ |
| 20 | Other benthic carnivorous fish | 3.79^c^ | 3.79^c^ | 3.79^c^ | 50.07^c^ | 50.07^c^ | 50.07^c^ |
| 21 | Red tjor-tjor | 3.70^c^ | 3.72^c^ | 3.75^c^ | 26.69^c^ | 34.69^c^ | 35.25^c^ |
| 22 | Pinky | 3.76^c^ | 3.76^c^ | 3.76^c^ | 45.05^c^ | 45.05^c^ | 45.05^c^ |
| 23 | Other benthopelagic fish | 4.10^c^ | 3.81^c^ | 4.47^c^ | 49.44^c^ | 45.12^c^ | 41.00^c^ |
| 24 | Small pelagic fish | 3.83 ^j^ | 3.83 ^j^ | 3.83 ^j^ | 23.15 ^j^ | 23.15 ^j^ | 23.15 ^j^ |
| 25 | Large pelagic fish | 3.16 ^j^ | 3.16 ^j^ | 3.16^j^ | 10.89^j^ | 10.89^j^ | 10.89^j^ |
| 26 | Skates and rays | 3.86^c^ | 3.86^c^ | 3.86^c^ | 41.53^c^ | 41.53^c^ | 41.53^c^ |
| 27 | Small benthic sharks | 3.03^c^ | 3.11^c^ | 3.18^c^ | 41.51^c^ | 50.56^c^ | 50.56^c^ |
| 28 | Large sharks | 3.24^h^ | 3.24^h^ | 3.24^h^ | 16.38^h^ | 17.91^h^ | 19.44^h^ |
| 29 | Cetaceans | 3.2^i^ | 3.2^i^ | 3.2^i^ | 12.80^l^ | 12.80^l^ | 12.80^l^ |
| 30 | Suspended POM | 8.2^a^ | 8.2^a^ | 8.2^a^ | 20.00^a^ | 20.00^a^ | 20.00^a^ |
| 31 | Sediment POM | 8.03^c^ | 10.19^c^ | 8.03^c^ | 57.84^c^ | 73.36^c^ | 57.84^c^ |
| 32 | DOM | 3.40^j^ | 3.40^j^ | 3.40^j^ | 44.00^m^ | 44.00^m^ | 44.00^m^ |

References: a: (1), b: (2); c: (3) and references therein; d: (4); e: (5); f: (6); g: (7); h: (8); i: (9); j: (10); k: (11); l: (12); m: (13).

References to S4 Table:

1. Diaz P, Raimbault F, Boudjellal B, Garcia N, Moutin T. Early spring phosphorus limitation of primary productivity in a NW Mediterranean coastal zone (Gulf of Lions). Mar Ecol Prog Ser. 2001;211:51–62.

2. Le Borgne R. Zooplankton production in the eastern tropical Atlantic Ocean: Net growth efficiency and P:B in terms of carbon, nitrogen, and phosphorus. Limnol Oceanogr. 1982;27(4):681–98.

3. Scharler UM, Ayers MJ, de Lecea AM, Fennessy ST, Pretorious M, Huggett JA, et al. Riverine influence determines nearshore heterogeneity of nutrient (C,N,P) content and stoichiometry in the KwaZulu-Natal Bight, South Africa. African J Mar Sci. 2016;38(Supplement):S193–4.

4. Beers J. Studies on the chemical composition of the major zooplankton in the Sargasso Sea of Bermuda. Limnol Oceanogr. 1966;11(4):520–8.

5. Newell R. An evaluation of the wet oxidation technique for use in determining the energy content of seston samples. Can J Fish Aquat Sci. 1982;39(10):1383–8.

6. Clarke A. Ecological stoichiometry in six species of Antarctic marine benthos. Mar Ecol Prog Ser [Internet]. 2008;369:25–37. Available from: http://www.int-res.com/prepress/m07670.html

7. Czamanski M, Nugraha A, Pondaven P, Lasbleiz M, Masson A, Caroff N, et al. Carbon, nitrogen and phosphorus elemental stoichiometry in aquacultured and wild-caught fish and consequences for pelagic nutrient dynamics. Mar Biol. 2011;158(12):2847–62.

8. Hussey NE, Brush J, McCarthy ID, Fisk AT. δ15N and δ13C diet-tissue discrimination factors for large sharks under semi-controlled conditions. Comp Biochem Physiol - A Mol Integr Physiol [Internet]. 2010;155(4):445–53. Available from: http://dx.doi.org/10.1016/j.cbpa.2009.09.023

9. Ruiz-Cooley RI, Gendron D, Aguinia S, Mesnick S, Carriquiry JD. Trophic relationships between sperm whales and jumbo squid using stable isotopes of C and Null hypothesis testing: problems, prevalence, and an alternative. Mar Ecol Ser. 2004;277:275–83.

10. Baird D, Ulanowicz RE, Boynton WR. Seasonal nitrogen dynamics in Chesapeake Bay: a network approach. Estuar Coast Shelf Sci. 1995;41(2):137–62.

11. Vink S, Atkinson MJ. High dissolved C : P excretion ratios for large benthic marine invertebrates. October. 1985;21:191–5.

12. Portnoy J. Gull contributions of phosphorus and nitrogen to a Cape Cod kettle pond. Hydrobiologia. 1990;202(1–2):61–9.

13. Baird D. Seasonal phosphorus dynamics in the Chesapeake Bay. Solomons, MD, USA; 1998.
