# Supplementary material for: Stoichiometric multitrophic networks reveal significance of land-sea interaction to ecosystem function in a subtropical nutrient-poor bight, South Africa
Source: PLoS One. 2019 Jan 7;14(1):e0210295. doi: 10.1371/journal.pone.0210295 (PMC6322777; doi:10.1371/journal.pone.0210295)
Supplement: S5 Table — Bold values represent biomasses estimated by Ecopath. (DOCX) [file pone.0210295.s005.docx]

S 5:

|  | Functional groups/species |  | Durban Eddy | | Thukela Mouth | | Richards Bay | |
| --- | --- | --- | --- | --- | --- | --- | --- | --- |
|  |  |  | Summer | Winter | Summer | Winter | Summer | Winter |
| 1 | Diatoms | C | 1.15 | 2.43 | 0.46 | 0.39 | 0.50 | 1.93 |
|  |  | N | 0.14 | 0.30 | 0.06 | 0.06 | 0.06 | 0.24 |
|  |  | P | 0.06 | 0.12 | 0.02 | 0.02 | 0.03 | 0.10 |
| 2 | Flagellates | C | 1.23 | 1.12 | 0.46 | 0.26 | 0.34 | 0.37 |
|  |  | N | 0.15 | 0.14 | 0.06 | 0.03 | 0.04 | 0.04 |
|  |  | P | 0.06 | 0.06 | 0.02 | 0.01 | 0.02 | 0.02 |
| 3 | Bacteria | C | 1.84 | 0.60 | 0.69 | 0.29 | 0.52 | 0.31 |
|  |  | N | 0.24 | 0.08 | 0.09 | 0.04 | 0.07 | 0.04 |
|  |  | P | 0.05 | 0.02 | 0.02 | 0.01 | 0.01 | 0.01 |
| 4 | Heterotrophic microplankton | C | **0.20** | **0.20** | **0.08** | **0.24** | **0.08** | **0.06** |
|  |  | N | 0.04 | 0.05 | 0.01 | 0.05 | 0.02 | 0.01 |
|  |  | P | <0.01 | 0.06 | <0.01 | <0.01 | <0.01 | <0.01 |
| 5 | Small copepods | C | 0.37 | 0.15 | 0.11 | 0.16 | 0.04 | 0.11 |
|  |  | N | 0.08 | 0.03 | 0.02 | 0.04 | 0.01 | 0.03 |
|  |  | P | <0.01 | <0.01 | <0.01 | <0.01 | <0.01 | <0.01 |
| 6 | Medium copepods | C | 0.17 | 0.13 | 0.06 | 0.12 | 0.05 | 0.05 |
|  |  | N | 0.04 | 0.03 | 0.01 | 0.03 | 0.01 | 0.01 |
|  |  | P | <0.01 | <0.01 | <0.01 | <0.01 | <0.01 | <0.01 |
| 7 | Large copepods | C | 0.07 | 0.15 | 0.03 | 0.13 | 0.03 | 0.10 |
|  |  | N | 0.02 | 0.03 | 0.01 | 0.03 | 0.01 | 0.02 |
|  |  | P | <0.01 | <0.01 | <0.01 | <0.01 | <0.01 | <0.01 |
| 8 | Other large zooplankton | C | 0.03 | 0.09 | 0.02 | 0.03 | 0.03 | 0.04 |
|  |  | N | 0.01 | 0.03 | 0.01 | 0.01 | 0.01 | 0.01 |
|  |  | P | <0.01 | <0.01 | <0.01 | <0.01 | <0.01 | <0.01 |
| 9 | Small macrobenthos | C | **3515.17** | **4751.11** | **1376.05** | **4392.53** | **1482.28** | **1064.64** |
|  |  | N | 752.71 | 1017.37 | 294.66 | 940.58 | 317.41 | 227.98 |
|  |  | P | 40.40 | 54.61 | 15.82 | 50.49 | 17.04 | 12.24 |
| 10 | Large suspension feeders | C | **2122.76** | **2658.02** | **1239.95** | **3858.49** | **435.90** | **586.82** |
|  |  | N | 496.33 | 621.49 | 289.92 | 902.17 | 101.92 | 137.21 |
|  |  | P | 29.54 | 36.98 | 17.25 | 53.69 | 6.06 | 8.16 |
| 11 | Echinoderms | C | **154.39** | **282.23** | 14.88 | 4.91 | 1.01 | 1.13 |
|  |  | N | 37.11 | 67.84 | 3.58 | 1.18 | 0.24 | 0.27 |
|  |  | P | 0.85 | 1.55 | 0.08 | 0.03 | 0.01 | 0.01 |
| 12 | Molluscs (non-cephalopod) | C | 138.3 | **271.33** | **128.92** | **363.66** | 1.22 | **4.22** |
|  |  | N | 40.28 | 79.02 | 37.54 | 105.91 | 0.35 | 1.23 |
|  |  | P | 3.43 | 6.73 | 3.20 | 9.02 | 0.03 | 0.10 |
| 13 | Prawn and shrimp | C | **335.05** | **470.08** | 0.10 | **0.33** | 0.59 | 2.67 |
|  |  | N | 102.94 | 144.42 | 0.03 | 0.10 | 0.18 | 0.82 |
|  |  | P | 25.23 | 35.40 | 0.01 | 0.02 | 0.04 | 0.20 |
| 14 | Large crustaceans | C | 88.01 | 83.26 | 24.08 | 2.92 | 8.08 | 48.11 |
|  |  | N | 27.23 | 25.76 | 7.45 | 0.90 | 2.50 | 14.88 |
|  |  | P | 6.30 | 5.96 | 1.72 | 0.21 | 0.58 | 3.44 |
| 15 | Cuttlefish | C | **720.60** | **606.01** | **104.07** | **318.03** | **385.78** | **79.60** |
|  |  | N | 209.67 | 176.33 | 33.57 | 102.59 | 112.25 | 23.16 |
|  |  | P | 51.35 | 43.18 | 8.76 | 26.75 | 27.49 | 5.67 |
| 16 | Other cephalopods | C | **233.29** | **400.96** | **67.25** | **412.35** | **39.64** | **31.33** |
|  |  | N | 69.49 | 119.43 | 20.03 | 122.82 | 11.81 | 9.33 |
|  |  | P | 20.98 | 36.06 | 6.05 | 37.09 | 3.56 | 2.82 |
| 17 | Flatfish | C | 130.33 | 254.13 | 66.29 | 239.37 | 34.77 | 150.93 |
|  |  | N | 40.37 | 78.71 | 2.053 | 74.14 | 10.77 | 46.75 |
|  |  | P | 7.91 | 15.42 | 4.02 | 14.53 | 2.73 | 11.86 |
| 18 | Gurnard | C | 336.31 | 287.76 | 146.72 | 568.66 | 5.55 | 179.14 |
|  |  | N | 102.75 | 87.92 | 44.83 | 173.74 | 1.70 | 54.73 |
|  |  | P | 19.29 | 16.50 | 8.42 | 32.62 | 0.44 | 14.06 |
| 19 | Lizardfish | C | 73.61 | 25.69 | 2.28 | 4.64 | 6.57 | 10.48 |
|  |  | N | 22.69 | 15.54 | 0.72 | 1.45 | 2.06 | 3.28 |
|  |  | P | 3.53 | 2.42 | 0.15 | 0.31 | 0.44 | 0.69 |
| 20 | Other benthic carnivorous fish | C | 52.75 | 255.94 | 88.64 | 291.77 | 44.06 | 26.20 |
|  |  | N | 16.53 | 8.05 | 27.32 | 89.92 | 13.58 | 19.78 |
|  |  | P | 3.50 | 1.70 | 4.25 | 13.99 | 2.11 | 3.08 |
| 21 | Red tjor-tjor | C | 83.21 | 169.73 | 137.44 | 137.44 | 22.55 | 22.55 |
|  |  | N | 26.03 | 80.07 | 43.00 | 43.00 | 7.06 | 7.06 |
|  |  | P | 6.58 | 20.24 | 10.87 | 10.87 | 1.78 | 1.78 |
| 22 | Pinky | C | **13.95** | **9.81** | 82.96 | 175.09 | 3.57 | 2.57 |
|  |  | N | 4.34 | 3.05 | 25.81 | 54.48 | 1.11 | 0.80 |
|  |  | P | 0.70 | 0.49 | 4.16 | 8.78 | 0.18 | 0.13 |
| 23 | Other benthopelagic fish | C | 232.27 | 169.73 | 30.17 | 266.46 | 345.60 | 26.20 |
|  |  | N | 71.22 | 47.92 | 8.52 | 75.22 | 97.56 | 7.40 |
|  |  | P | 16.33 | 10.98 | 1.95 | 17.24 | 22.36 | 1.70 |
| 24 | Small pelagic fish | C | **83.21** | **133.23** | **68.39** | **59.60** | **48.38** | **68.89** |
|  |  | N | 21.72 | 34.82 | 17.87 | 15.58 | 12.64 | 18.00 |
|  |  | P | 3.59 | 5.76 | 2.95 | 2.57 | 2.09 | 2.98 |
| 25 | Large pelagic fish | C | **5.64** | **5.64** | **9.81** | **9.71** | **9.14** | **9.14** |
|  |  | N | 1.78 | 1.78 | 3.10 | 3.07 | 2.89 | 2.89 |
|  |  | P | 0.52 | 0.52 | 0.90 | 0.89 | 0.84 | 0.84 |
| 26 | Skates and rays | C | **15.63** | **15.63** | 118.73 | 970.68 | **17.13** | **17.13** |
|  |  | N | 4.96 | 4.96 | 41.02 | 335.32 | 5.44 | 5.44 |
|  |  | P | 1.02 | 1.02 | 7.75 | 63.39 | 1.12 | 1.12 |
| 27 | Small benthic sharks | C | 238.45 | 766.05 | 4.04 | 43.20 | **7.60** | **7.60** |
|  |  | N | 87.82 | 282.11 | 1.49 | 15.91 | 2.80 | 2.80 |
|  |  | P | 14.56 | 46.78 | 0.23 | 2.41 | 0.39 | 0.39 |
| 28 | Large sharks | C | 18.00 | 18.00 | 18.00 | 18.00 | 18.00 | 18.00 |
|  |  | N | 5.56 | 5.55 | 5.56 | 5.56 | 5.56 | 5.56 |
|  |  | P | 1.01 | 1.01 | 1.01 | 1.01 | 0.93 | 0.93 |
| 29 | Cetaceans | C | n/a | n/a | **7.13** | **7.05** | **6.64** | **6.64** |
|  |  | N | n/a | n/a | 2.23 | 2.20 | 2.08 | 2.08 |
|  |  | P | n/a | n/a | 0.56 | 0.55 | 0.52 | 0.52 |
| 30 | Suspended POM | C | 7.28 | 7.28 | 1.33 | 0.19 | 0.33 | 0.33 |
|  |  | N | 0.89 | 0.89 | 0.16 | 0.02 | 0.04 | 0.04 |
|  |  | P | 0.36 | 0.36 | 0.07 | 0.01 | 0.02 | 0.02 |
| 31 | Sediment POM | C | 378.60 | 378.60 | 1890.00 | 1890.00 | 378.55 | 378.55 |
|  |  | N | 47.12 | 47.12 | 185.92 | 185.92 | 47.12 | 47.12 |
|  |  | P | 6.54 | 6.54 | 25.82 | 25.82 | 6.54 | 6.54 |
| 32 | DOM | C | 378.60 | 378.60 | 271.20 | 271.20 | 271.20 | 271.20 |
|  |  | N | 0.89 | 0.89 | 4.34 | 4.34 | 4.34 | 4.34 |
|  |  | P | 0.36 | 0.36 | 0.34 | 0.34 | 0.34 | 0.34 |
| 33 | DIM | C | 28600.00 | 10641.66 | 2796.53 | 1375.27 | 1360.00 | 1190.00 |
|  |  | N | 11.7 | 13.68 | 1.15 | 0.57 | 0.57 | 0.49 |
|  |  | P | 2.11 | 3.62 | 0.35 | 0.45 | 0.39 | 0.24 |
