# Supplementary material for: Stoichiometric multitrophic networks reveal significance of land-sea interaction to ecosystem function in a subtropical nutrient-poor bight, South Africa
Source: PLoS One. 2019 Jan 7;14(1):e0210295. doi: 10.1371/journal.pone.0210295 (PMC6322777; doi:10.1371/journal.pone.0210295)
Supplement: S6 Table — DE: Durban Eddy, TM: Thukela Mouth, RB: Richard Bay. S: summer, w: winter. LN: Limiting Nutrient. C: carbon, N: nitrogen, P: phosphorus. (DOCX) [file pone.0210295.s006.docx]

S6:

|  | Node |  | Sensitivity values | | | | Proportion Consumption to Biomass | | | |
| --- | --- | --- | --- | --- | --- | --- | --- | --- | --- | --- |
|  | # | Node | C | N | P | LN | C | N | P | LN |
| DEs | 1 | Dia toms | -1.51E+03 | -9.31E+02 | -8.86E+02 | P | 7.45E+02 | 4.91E+02 | 4.69E+02 | P |
|  | 2 | Fla gellates | -1.44E+03 | -7.52E+02 | -8.31E+02 | P | 7.81E+02 | 4.01E+02 | 4.00E+02 | P |
|  | 3 | Bac teria | -3.75E+02 | -4.78E+02 | -7.58E+02 | C | 2.15E+02 | 1.66E+02 | 1.64E+02 | P |
|  | 4 | HM plankton | -3.75E+02 | -1.89E+02 | -7.48E+02 | P | 2.15E+02 | 1.22E+02 | 1.21E+02 | P |
|  | 5 | Sma ll copepods | -2.75E+02 | -1.51E+02 | -7.41E+02 | N | 1.65E+02 | 9.17E+01 | 4.43E+02 | N |
|  | 6 | Med ium copepods | -2.75E+02 | -1.43E+02 | -4.68E+02 | N | 1.65E+02 | 1.03E+02 | 4.07E+02 | N |
|  | 7 | Lar ge copepods | -2.74E+02 | -1.28E+02 | -1.87E+02 | N | 1.65E+02 | 9.92E+01 | 3.98E+02 | N |
|  | 8 | Oth er lg zooplan kton | -8.50E+01 | -2.78E+01 | -1.65E+02 | N | 2.00E+01 | 1.05E+01 | 1.79E+01 | N |
|  | 9 | Sm macrobenthos | 1.54E+01 | 3.48E+01 | 2.03E+01 | N | 7.02E+01 | 4.11E+01 | 1.10E+02 | N |
|  | 10 | Lg suspension fe eder | 3.30E+01 | 3.91E+01 | 2.66E+01 | N | 5.00E-01 | 2.85E-01 | 1.61E+00 | N |
|  | 11 | Ech inoderm | 3.54E+01 | 4.10E+01 | 3.18E+01 | N | 4.00E+00 | 2.71E+00 | 1.44E+01 | N |
|  | 12 | Mol lusc | 3.75E+01 | 4.31E+01 | 3.86E+01 | N | 8.20E+00 | 4.26E+00 | 8.42E+00 | N |
|  | 13 | Pra wn & shrimp | 3.90E+01 | 4.40E+01 | 4.29E+01 | P | 6.10E+00 | 4.42E+00 | 2.74E+00 | P |
|  | 14 | Lar ge crustacean | 4.08E+01 | 4.41E+01 | 4.39E+01 | P | 1.00E+01 | 7.22E+00 | 1.87E+00 | P |
|  | 15 | Cut tlefish | 4.16E+01 | 4.45E+01 | 4.56E+01 | P | 3.50E+00 | 2.75E+00 | 1.25E+00 | P |
|  | 16 | Oth er cephalopod | 4.18E+01 | 4.48E+01 | 4.74E+01 | P | 3.90E+00 | 3.13E+00 | 2.20E+00 | P |
|  | 17 | Fla tfish | 4.30E+01 | 4.53E+01 | 4.86E+01 | P | 6.80E+00 | 5.05E+00 | 1.99E+00 | P |
|  | 18 | Gur nard | 4.30E+01 | 4.66E+01 | 4.91E+01 | P | 3.50E+00 | 2.82E+00 | 2.08E+00 | P |
|  | 19 | Oth er bc fish | 4.32E+01 | 4.69E+01 | 5.00E+01 | N | 6.20E+00 | 5.31E+00 | 5.76E+00 | N |
|  | 20 | Liz ardfish | 4.58E+01 | 4.70E+01 | 5.10E+01 | P | 7.30E+00 | 6.15E+00 | 4.91E+00 | P |
|  | 21 | Red tjor-tjor | 4.74E+01 | 4.92E+01 | 5.11E+01 | P | 6.90E+00 | 5.72E+00 | 3.16E+00 | P |
|  | 22 | Pin ky | 4.76E+01 | 4.98E+01 | 5.13E+01 | P | 4.80E+00 | 4.19E+00 | 4.02E+00 | P |
|  | 23 | Oth er bp | 4.84E+01 | 4.99E+01 | 5.14E+01 | P | 6.20E+00 | 5.48E+00 | 3.40E+00 | P |
|  | 24 | Sm pelagic fish | 4.84E+01 | 5.00E+01 | 5.16E+01 | N | 1.12E+01 | 5.68E+00 | 1.18E+01 | N |
|  | 25 | Lg pelagic fish | 4.92E+01 | 5.10E+01 | 5.17E+01 | P | 8.98E+00 | 8.15E+00 | 6.26E+00 | P |
|  | 26 | Ska tes and rays | 5.02E+01 | 5.11E+01 | 5.18E+01 | P | 2.60E+00 | 2.19E+00 | 1.79E+00 | P |
|  | 27 | Sm benthic shark | 5.19E+01 | 5.19E+01 | 5.22E+01 | P | 3.10E+00 | 2.18E+00 | 2.18E+00 | P |
|  | 28 | Lar ge sharks | 5.44E+01 | 5.49E+01 | 5.29E+01 | C | 1.75E+00 | 1.77E+00 | 1.90E+00 | C |
| DEw | 1 | Diatoms | 4.06E+01 | 4.07E+01 | 4.07E+01 | P | 7.45E+02 | 4.15E+02 | 4.14E+02 | P |
|  | 2 | Flagellates | 4.06E+01 | 4.07E+01 | 4.07E+01 | P | 7.81E+02 | 5.57E+02 | 5.53E+02 | P |
|  | 3 | Bacteria | -3.82E+02 | -1.24E+01 | -3.72E+02 | N | 2.15E+02 | 1.56E+02 | 2.16E+02 | N |
|  | 4 | HM plankton | -5.22E+02 | -2.71E+02 | -1.07E+00 | P | 2.92E+02 | 1.66E+02 | 1.53E+02 | P |
|  | 5 | Small copepods | -7.77E+00 | 1.38E+01 | -9.04E+01 | N | 1.65E+02 | 9.19E+01 | 4.50E+02 | N |
|  | 6 | Medium copepods | -9.42E+01 | -3.86E+01 | -3.22E+02 | N | 1.65E+02 | 1.03E+02 | 4.08E+02 | N |
|  | 7 | Large copepods | -9.74E+01 | -3.84E+01 | -3.03E+02 | N | 1.65E+02 | 9.90E+01 | 3.84E+02 | N |
|  | 8 | Other lg zooplankton | 1.46E+01 | 2.59E+01 | 1.36E+01 | N | 2.00E+01 | 1.18E+01 | 1.87E+01 | N |
|  | 9 | Sm macrobenthos | -6.26E+01 | -2.14E+01 | -1.24E+02 | N | 7.02E+01 | 4.02E+01 | 9.65E+01 | N |
|  | 10 | Lg suspension feeder | 3.99E+01 | 4.03E+01 | 3.76E+01 | N | 5.00E-01 | 2.84E-01 | 1.75E+00 | N |
|  | 11 | Echinoderm | 3.43E+01 | 3.64E+01 | 1.73E+01 | N | 4.00E+00 | 2.71E+00 | 1.44E+01 | N |
|  | 12 | Mollusc | 2.67E+01 | 3.36E+01 | 2.58E+01 | N | 8.20E+00 | 4.27E+00 | 8.42E+00 | N |
|  | 13 | Prawn & shrimp | 3.18E+01 | 3.42E+01 | 3.69E+01 | P | 6.10E+00 | 4.45E+00 | 2.45E+00 | P |
|  | 14 | Large crustacean | 2.52E+01 | 2.95E+01 | 3.78E+01 | P | 1.00E+01 | 7.22E+00 | 1.87E+00 | P |
|  | 15 | Cuttlefish | 3.58E+01 | 3.69E+01 | 3.94E+01 | P | 3.50E+00 | 2.75E+00 | 1.07E+00 | P |
|  | 16 | Other cephalopod | 3.59E+01 | 3.70E+01 | 3.95E+01 | P | 3.90E+00 | 3.12E+00 | 1.53E+00 | P |
|  | 17 | Flatfish | 3.07E+01 | 3.33E+01 | 3.79E+01 | P | 6.80E+00 | 5.04E+00 | 1.95E+00 | P |
|  | 18 | Gurnard | 3.60E+01 | 3.70E+01 | 3.82E+01 | P | 3.50E+00 | 2.80E+00 | 2.05E+00 | P |
|  | 19 | Other bc fish | 3.22E+01 | 3.35E+01 | 3.32E+01 | N | 6.20E+00 | 5.32E+00 | 5.77E+00 | N |
|  | 20 | Lizardfish | 3.07E+01 | 3.23E+01 | 3.43E+01 | P | 7.30E+00 | 6.15E+00 | 4.91E+00 | P |
|  | 21 | Red tjor-tjor | 3.03E+01 | 3.27E+01 | 3.84E+01 | P | 6.90E+00 | 5.27E+00 | 1.59E+00 | P |
|  | 22 | Pinky | 3.31E+01 | 3.41E+01 | 3.48E+01 | P | 4.80E+00 | 4.19E+00 | 4.02E+00 | P |
|  | 23 | Other bp | 3.23E+01 | 3.34E+01 | 3.66E+01 | P | 6.20E+00 | 5.48E+00 | 3.40E+00 | P |
|  | 24 | Sm pelagic fish | 3.73E+01 | 3.85E+01 | 3.83E+01 | N | 1.12E+01 | 5.65E+00 | 1.19E+01 | N |
|  | 25 | Lg pelagic fish | 3.08E+01 | 3.17E+01 | 3.37E+01 | P | 8.98E+00 | 8.13E+00 | 6.27E+00 | P |
|  | 26 | Skates and rays | 3.72E+01 | 3.78E+01 | 3.87E+01 | P | 2.60E+00 | 2.19E+00 | 1.79E+00 | P |
|  | 27 | Sm benthic shark | 3.69E+01 | 3.81E+01 | 3.84E+01 | P | 3.10E+00 | 2.19E+00 | 2.21E+00 | N |
|  | 28 | Large sharks | 3.85E+01 | 3.84E+01 | 3.83E+01 | C | 1.74E+00 | 1.77E+00 | 1.90E+00 | C |
| TMs | 1 | Diatoms | -1.63E+03 | -1.28E+03 | -1.24E+03 | P | 8.38E+02 | 6.62E+02 | 6.43E+02 | P |
|  | 2 | Flagellates | -2.74E+03 | -1.77E+03 | -1.68E+03 | P | 1.39E+03 | 9.13E+02 | 8.66E+02 | P |
|  | 3 | Bacteria | -3.79E+02 | -3.19E+02 | -3.19E+02 | N | 2.15E+02 | 2.15E+02 | 2.15E+02 | P |
|  | 4 | HM plankton | -3.80E+02 | -1.67E+02 | -1.72E+02 | N | 2.15E+02 | 1.09E+02 | 1.11E+02 | N |
|  | 5 | Small copepods | -2.79E+02 | -1.34E+02 | -8.30E+02 | N | 1.65E+02 | 9.20E+01 | 4.40E+02 | N |
|  | 6 | Medium copepods | -2.79E+02 | -1.56E+02 | -7.73E+02 | N | 1.65E+02 | 1.03E+02 | 4.13E+02 | N |
|  | 7 | Large copepods | -2.79E+02 | -1.46E+02 | -7.57E+02 | N | 1.65E+02 | 9.86E+01 | 4.04E+02 | N |
|  | 8 | Other lg zooplankton | 1.06E+01 | 2.96E+01 | 1.13E+01 | N | 2.00E+01 | 1.03E+01 | 1.96E+01 | N |
|  | 9 | Sm macrobenthos | -8.98E+01 | -2.68E+01 | -1.30E+02 | N | 7.02E+01 | 2.15E+01 | 5.10E+01 | N |
|  | 10 | Lg suspension feeder | 4.96E+01 | 4.98E+01 | 4.72E+01 | N | 5.00E-01 | 5.39E-01 | 1.69E+00 | C |
|  | 11 | Echinoderm | 4.26E+01 | 4.56E+01 | 2.44E+01 | N | 4.00E+00 | 2.48E+00 | 1.31E+01 | N |
|  | 12 | Mollusc | 3.42E+01 | 4.28E+01 | 3.48E+01 | N | 8.20E+00 | 3.91E+00 | 7.91E+00 | N |
|  | 13 | Prawn & shrimp | 3.84E+01 | 4.52E+01 | 4.79E+01 | P | 6.10E+00 | 2.69E+00 | 1.26E+00 | P |
|  | 14 | Large crustacean | 3.06E+01 | 3.79E+01 | 4.70E+01 | P | 1.00E+01 | 6.32E+00 | 1.80E+00 | P |
|  | 15 | Cuttlefish | 4.36E+01 | 4.56E+01 | 4.83E+01 | P | 3.50E+00 | 2.48E+00 | 1.15E+00 | P |
|  | 16 | Other cephalopod | 4.28E+01 | 4.45E+01 | 4.65E+01 | P | 3.90E+00 | 3.01E+00 | 2.02E+00 | P |
|  | 17 | Flatfish | 3.70E+01 | 4.08E+01 | 4.74E+01 | P | 6.80E+00 | 4.90E+00 | 1.58E+00 | P |
|  | 18 | Gurnard | 4.36E+01 | 4.53E+01 | 4.82E+01 | P | 3.50E+00 | 2.65E+00 | 1.18E+00 | P |
|  | 19 | Other bc fish | 3.82E+01 | 4.11E+01 | 4.41E+01 | P | 6.20E+00 | 4.77E+00 | 3.26E+00 | P |
|  | 20 | Lizardfish | 3.60E+01 | 3.86E+01 | 4.19E+01 | P | 7.30E+00 | 6.01E+00 | 4.34E+00 | P |
|  | 21 | Red tjor-tjor | 3.68E+01 | 4.04E+01 | 4.81E+01 | P | 6.90E+00 | 5.10E+00 | 1.25E+00 | P |
|  | 22 | Pinky | 4.10E+01 | 4.35E+01 | 4.71E+01 | P | 4.80E+00 | 3.55E+00 | 1.74E+00 | P |
|  | 23 | Other bp | 3.82E+01 | 4.02E+01 | 4.60E+01 | P | 6.20E+00 | 5.18E+00 | 2.31E+00 | P |
|  | 24 | Sm pelagic fish | 2.82E+01 | 3.93E+01 | 2.67E+01 | N | 1.12E+01 | 5.66E+00 | 1.19E+01 | N |
|  | 25 | Lg pelagic fish | 3.26E+01 | 3.55E+01 | 4.08E+01 | P | 8.98E+00 | 7.55E+00 | 4.87E+00 | P |
|  | 26 | Skates and rays | 4.54E+01 | 4.70E+01 | 4.75E+01 | P | 2.60E+00 | 1.81E+00 | 1.55E+00 | P |
|  | 27 | Sm benthic shark | 4.44E+01 | 4.60E+01 | 4.46E+01 | N | 3.10E+00 | 2.31E+00 | 3.00E+00 | N |
|  | 28 | Large sharks | 4.71E+01 | 4.71E+01 | 4.66E+01 | N | 1.75E+00 | 1.74E+00 | 1.99E+00 | N |
|  | 29 | Cetaceans | 3.06E+01 | 3.22E+01 | 3.49E+01 | P | 1.00E+01 | 9.21E+00 | 7.84E+00 | P |
| TMw | 1 | Diatoms | -1.63E+03 | -1.43E+03 | -1.39E+03 | P | 8.39E+02 | 7.48E+02 | 3.50E+02 | P |
|  | 2 | Flagellates | -2.75E+03 | -2.41E+03 | -2.37E+03 | P | 1.39E+03 | 1.25E+03 | 6.25E+02 | P |
|  | 3 | Bacteria | -4.12E+02 | -3.23E+02 | -3.21E+02 | P | 1.80E+02 | 1.14E+02 | 1.14E+02 | P |
|  | 4 | HM plankton | -3.80E+02 | -1.69E+02 | -9.88E+02 | N | 2.12E+02 | 1.09E+02 | 5.18E+02 | N |
|  | 5 | Small copepods | -2.82E+02 | -1.36E+02 | -8.56E+02 | N | 1.65E+02 | 9.20E+01 | 4.52E+02 | N |
|  | 6 | Medium copepods | -2.82E+02 | -1.59E+02 | -7.87E+02 | N | 1.65E+02 | 1.03E+02 | 4.18E+02 | N |
|  | 7 | Large copepods | -2.82E+02 | -1.51E+02 | -7.44E+02 | N | 1.65E+02 | 9.93E+01 | 3.95E+02 | N |
|  | 8 | Other lg zooplankton | 7.95E+00 | 2.68E+01 | 1.00E+01 | N | 2.00E+01 | 1.05E+01 | 1.91E+01 | N |
|  | 9 | Sm macrobenthos | -9.24E+01 | -2.17E+01 | -8.91E+01 | N | 7.01E+01 | 3.48E+01 | 9.12E+01 | N |
|  | 10 | Lg suspension feeder | 4.69E+01 | 4.74E+01 | 4.44E+01 | N | 4.98E-01 | 2.77E-01 | 1.75E+00 | N |
|  | 11 | Echinoderm | 3.99E+01 | 4.30E+01 | 2.20E+01 | N | 4.00E+00 | 2.46E+00 | 1.30E+01 | N |
|  | 12 | Mollusc | 3.15E+01 | 4.02E+01 | 3.22E+01 | N | 8.19E+00 | 3.89E+00 | 7.89E+00 | N |
|  | 13 | Prawn & shrimp | 3.57E+01 | 4.26E+01 | 4.52E+01 | P | 6.09E+00 | 2.69E+00 | 1.40E+00 | P |
|  | 14 | Large crustacean | 2.79E+01 | 3.54E+01 | 4.44E+01 | P | 9.99E+00 | 6.25E+00 | 1.75E+00 | P |
|  | 15 | Cuttlefish | 4.09E+01 | 4.31E+01 | 4.56E+01 | P | 3.50E+00 | 2.42E+00 | 1.15E+00 | P |
|  | 16 | Other cephalopod | 4.01E+01 | 4.19E+01 | 4.39E+01 | P | 3.91E+00 | 3.02E+00 | 2.03E+00 | P |
|  | 17 | Flatfish | 3.43E+01 | 3.83E+01 | 4.49E+01 | P | 6.79E+00 | 4.82E+00 | 1.51E+00 | P |
|  | 18 | Gurnard | 4.09E+01 | 4.27E+01 | 4.57E+01 | P | 3.50E+00 | 2.61E+00 | 1.14E+00 | P |
|  | 19 | Other bc fish | 3.55E+01 | 3.85E+01 | 4.16E+01 | P | 6.21E+00 | 4.72E+00 | 3.17E+00 | P |
|  | 20 | Lizardfish | 3.33E+01 | 3.65E+01 | 4.09E+01 | P | 7.29E+00 | 5.70E+00 | 3.52E+00 | P |
|  | 21 | Red tjor-tjor | 3.41E+01 | 3.78E+01 | 4.55E+01 | P | 6.90E+00 | 5.06E+00 | 1.24E+00 | P |
|  | 22 | Pinky | 3.83E+01 | 4.09E+01 | 4.45E+01 | P | 4.80E+00 | 3.52E+00 | 1.73E+00 | P |
|  | 23 | Other bp | 3.56E+01 | 3.77E+01 | 4.50E+01 | P | 6.18E+00 | 5.13E+00 | 1.45E+00 | P |
|  | 24 | Sm pelagic fish | 2.55E+01 | 3.66E+01 | 2.42E+01 | N | 1.12E+01 | 5.67E+00 | 6.10E+00 | N |
|  | 25 | Lg pelagic fish | 3.00E+01 | 3.13E+01 | 3.41E+01 | P | 8.98E+00 | 8.31E+00 | 6.91E+00 | P |
|  | 26 | Skates and rays | 4.27E+01 | 4.41E+01 | 4.49E+01 | P | 2.60E+00 | 1.94E+00 | 1.54E+00 | P |
|  | 27 | Sm benthic shark | 4.17E+01 | 4.36E+01 | 4.34E+01 | N | 3.10E+00 | 2.15E+00 | 2.28E+00 | N |
|  | 28 | Large sharks | 4.44E+01 | 4.45E+01 | 4.39E+01 | N | 1.75E+00 | 1.74E+00 | 2.00E+00 | N |
|  | 29 | Cetaceans | 2.79E+01 | 2.92E+01 | 2.69E+01 | N | 1.00E+01 | 9.38E+00 | 1.05E+01 | N |
| RBs | 1 | Diatoms | -1.42E+03 | -1.10E+03 | -8.47E+02 | P | 7.44E+02 | 5.88E+02 | 4.57E+02 | P |
|  | 2 | Flagellates | -1.50E+03 | -1.20E+03 | -1.16E+03 | P | 7.82E+02 | 6.33E+02 | 6.13E+02 | P |
|  | 3 | Bacteria | -3.62E+02 | -3.09E+02 | -3.08E+02 | P | 2.15E+02 | 2.15E+02 | 2.14E+02 | P |
|  | 4 | HM plankton | -3.62E+02 | -1.49E+02 | -1.48E+02 | P | 2.15E+02 | 1.09E+02 | 1.08E+02 | P |
|  | 5 | Small copepods | -2.62E+02 | -8.97E+01 | -6.84E+02 | N | 1.65E+02 | 7.90E+01 | 3.76E+02 | N |
|  | 6 | Medium copepods | -2.61E+02 | -1.33E+02 | -7.14E+02 | N | 1.65E+02 | 1.01E+02 | 3.92E+02 | N |
|  | 7 | Large copepods | -2.62E+02 | -1.32E+02 | -6.55E+02 | N | 1.65E+02 | 1.00E+02 | 3.62E+02 | N |
|  | 8 | Other lg zooplankton | 2.82E+01 | 4.64E+01 | 2.98E+01 | N | 2.00E+01 | 1.10E+01 | 1.98E+01 | N |
|  | 9 | Sm macrobenthos | -7.22E+01 | -1.62E+01 | -1.52E+02 | N | 7.02E+01 | 4.22E+01 | 8.38E+01 | N |
|  | 10 | Lg suspension feeder | 6.71E+01 | 6.76E+01 | 6.50E+01 | N | 5.00E-01 | 2.64E-01 | 1.56E+00 | N |
|  | 11 | Echinoderm | 6.01E+01 | 6.27E+01 | 3.92E+01 | N | 4.00E+00 | 2.70E+00 | 1.45E+01 | N |
|  | 12 | Mollusc | 5.17E+01 | 5.96E+01 | 5.12E+01 | N | 8.20E+00 | 4.27E+00 | 8.51E+00 | N |
|  | 13 | Prawn & shrimp | 5.60E+01 | 6.21E+01 | 6.50E+01 | P | 6.10E+00 | 3.05E+00 | 1.59E+00 | P |
|  | 14 | Large crustacean | 4.81E+01 | 5.52E+01 | 6.44E+01 | P | 1.00E+01 | 6.46E+00 | 1.90E+00 | P |
|  | 15 | Cuttlefish | 6.12E+01 | 6.27E+01 | 6.59E+01 | P | 3.49E+00 | 2.72E+00 | 1.14E+00 | P |
|  | 16 | Other cephalopod | 6.04E+01 | 6.21E+01 | 6.41E+01 | P | 3.89E+00 | 3.01E+00 | 2.02E+00 | P |
|  | 17 | Flatfish | 5.45E+01 | 5.86E+01 | 6.57E+01 | P | 6.81E+00 | 4.77E+00 | 1.23E+00 | P |
|  | 18 | Gurnard | 6.11E+01 | 6.21E+01 | 6.40E+01 | P | 3.50E+00 | 3.05E+00 | 2.09E+00 | P |
|  | 19 | Other bc fish | 5.57E+01 | 5.87E+01 | 6.13E+01 | P | 6.21E+00 | 4.70E+00 | 3.41E+00 | P |
|  | 20 | Lizardfish | 5.35E+01 | 5.69E+01 | 6.12E+01 | P | 7.31E+00 | 5.62E+00 | 3.47E+00 | P |
|  | 21 | Red tjor-tjor | 5.43E+01 | 5.61E+01 | 5.82E+01 | P | 6.90E+00 | 6.01E+00 | 4.96E+00 | P |
|  | 22 | Pinky | 5.85E+01 | 6.09E+01 | 6.41E+01 | P | 4.80E+00 | 3.61E+00 | 2.00E+00 | P |
|  | 23 | Other bp | 5.57E+01 | 5.82E+01 | 6.44E+01 | P | 6.21E+00 | 4.96E+00 | 1.88E+00 | P |
|  | 24 | Sm pelagic fish | 4.57E+01 | 5.69E+01 | 4.43E+01 | N | 1.12E+01 | 5.65E+00 | 1.19E+01 | N |
|  | 25 | Lg pelagic fish | 5.02E+01 | 5.19E+01 | 5.54E+01 | P | 8.98E+00 | 8.11E+00 | 6.38E+00 | P |
|  | 26 | Skates and rays | 6.30E+01 | 6.39E+01 | 6.48E+01 | P | 2.60E+00 | 2.12E+00 | 1.67E+00 | P |
|  | 27 | Sm benthic shark | 6.19E+01 | 6.38E+01 | 6.28E+01 | N | 3.10E+00 | 2.18E+00 | 2.70E+00 | N |
|  | 28 | Large sharks | 6.46E+01 | 6.47E+01 | 6.39E+01 | N | 1.75E+00 | 1.72E+00 | 2.14E+00 | N |
|  | 29 | Cetaceans | 4.81E+01 | 5.01E+01 | 5.27E+01 | P | 1.00E+01 | 9.00E+00 | 7.75E+00 | P |
| RBw | 1 | Diatoms | -1.43E+03 | -6.44E+02 | -6.39E+02 | P | 7.44E+02 | 3.52E+02 | 3.50E+02 | P |
|  | 2 | Flagellates | -1.50E+03 | -8.64E+02 | -8.63E+02 | P | 7.80E+02 | 4.61E+02 | 4.61E+02 | P |
|  | 3 | Bacteria | -3.69E+02 | -3.13E+02 | -3.12E+02 | P | 2.15E+02 | 2.15E+02 | 2.14E+02 | P |
|  | 4 | HM plankton | -3.70E+02 | -1.56E+02 | -1.61E+02 | N | 2.16E+02 | 1.09E+02 | 1.11E+02 | N |
|  | 5 | Small copepods | -2.69E+02 | -9.63E+01 | -6.86E+02 | N | 1.65E+02 | 7.86E+01 | 3.73E+02 | N |
|  | 6 | Medium copepods | -2.69E+02 | -1.42E+02 | -7.56E+02 | N | 1.65E+02 | 1.02E+02 | 4.09E+02 | N |
|  | 7 | Large copepods | -2.69E+02 | -1.44E+02 | -7.48E+02 | N | 1.65E+02 | 1.03E+02 | 4.05E+02 | N |
|  | 8 | Other lg zooplankton | 2.10E+01 | 3.89E+01 | 2.42E+01 | N | 2.00E+01 | 1.10E+01 | 1.86E+01 | N |
|  | 9 | Sm macrobenthos | -7.95E+01 | -2.35E+01 | -1.60E+02 | N | 7.02E+01 | 4.22E+01 | 1.10E+02 | N |
|  | 10 | Lg suspension feeder | 6.00E+01 | 6.04E+01 | 5.76E+01 | N | 5.00E-01 | 2.62E-01 | 1.70E+00 | N |
|  | 11 | Echinoderm | 5.30E+01 | 5.55E+01 | 3.21E+01 | N | 4.00E+00 | 2.71E+00 | 1.45E+01 | N |
|  | 12 | Mollusc | 4.46E+01 | 5.24E+01 | 4.40E+01 | N | 8.20E+00 | 4.27E+00 | 8.48E+00 | N |
|  | 13 | Prawn & shrimp | 4.88E+01 | 5.48E+01 | 5.78E+01 | P | 6.10E+00 | 3.05E+00 | 1.60E+00 | P |
|  | 14 | Large crustacean | 4.10E+01 | 4.80E+01 | 5.72E+01 | P | 9.98E+00 | 6.46E+00 | 1.90E+00 | P |
|  | 15 | Cuttlefish | 5.40E+01 | 5.55E+01 | 5.87E+01 | P | 3.50E+00 | 2.75E+00 | 1.14E+00 | P |
|  | 16 | Other cephalopod | 5.32E+01 | 5.49E+01 | 5.69E+01 | P | 3.90E+00 | 3.03E+00 | 2.02E+00 | P |
|  | 17 | Flatfish | 4.74E+01 | 5.14E+01 | 5.86E+01 | P | 6.80E+00 | 4.80E+00 | 1.19E+00 | P |
|  | 18 | Gurnard | 5.40E+01 | 5.53E+01 | 5.80E+01 | P | 3.50E+00 | 2.83E+00 | 1.46E+00 | P |
|  | 19 | Other bc fish | 4.86E+01 | 5.15E+01 | 5.42E+01 | P | 6.20E+00 | 4.72E+00 | 3.38E+00 | P |
|  | 20 | Lizardfish | 4.64E+01 | 4.93E+01 | 5.28E+01 | P | 7.31E+00 | 5.82E+00 | 4.07E+00 | P |
|  | 21 | Red tjor-tjor | 4.72E+01 | 5.12E+01 | 5.80E+01 | P | 6.90E+00 | 4.90E+00 | 1.46E+00 | P |
|  | 22 | Pinky | 5.13E+01 | 5.37E+01 | 5.69E+01 | P | 4.80E+00 | 3.62E+00 | 2.01E+00 | P |
|  | 23 | Other bp | 4.86E+01 | 5.08E+01 | 5.60E+01 | P | 6.21E+00 | 5.06E+00 | 2.47E+00 | P |
|  | 24 | Sm pelagic fish | 3.86E+01 | 5.04E+01 | 3.51E+01 | N | 1.12E+01 | 5.26E+00 | 1.29E+01 | N |
|  | 25 | Lg pelagic fish | 4.30E+01 | 4.57E+01 | 5.13E+01 | P | 8.98E+00 | 7.61E+00 | 4.84E+00 | P |
|  | 26 | Skates and rays | 5.58E+01 | 5.67E+01 | 5.76E+01 | P | 2.60E+00 | 2.12E+00 | 1.67E+00 | P |
|  | 27 | Sm benthic shark | 5.48E+01 | 5.65E+01 | 5.52E+01 | N | 3.10E+00 | 2.23E+00 | 2.88E+00 | N |
|  | 28 | Large sharks | 5.75E+01 | 5.75E+01 | 5.67E+01 | N | 1.75E+00 | 1.72E+00 | 2.14E+00 | N |
|  | 29 | Cetaceans | 4.10E+01 | 4.29E+01 | 4.54E+01 | P | 1.00E+01 | 9.02E+00 | 7.77E+00 | P |
